# Dinaciclib, a cyclin-dependent kinase inhibitor, suppresses cholangiocarcinoma growth by targeting CDK2/5/9

**DOI:** 10.1038/s41598-020-75578-5

**Published:** 2020-10-28

**Authors:** Hera Saqub, Hannah Proetsch-Gugerbauer, Vladimir Bezrookove, Mehdi Nosrati, Edith M. Vaquero, David de Semir, Ryan J. Ice, Sean McAllister, Liliana Soroceanu, Mohammed Kashani-Sabet, Robert Osorio, Altaf A. Dar

**Affiliations:** California Pacific Medical Center Research Institute, 475 Brannan St, Suite 130, San Francisco, CA 94107 USA

**Keywords:** Cancer, Gastrointestinal cancer, Biliary tract cancer

## Abstract

Cholangiocarcinoma (CCA) is a highly invasive cancer, diagnosed at an advanced stage, and refractory to surgical intervention and chemotherapy. Cyclin-dependent kinases (CDKs) regulate cell cycle progression and transcriptional processes, and are considered potential therapeutic targets for cancer. Dinaciclib is a small molecule multi-CDK inhibitor targeting CDK 2/5/9. In this study, the therapeutic efficacy of dinaciclib was assessed using patient-derived xenograft cells (PDXC) and CCA cell lines. Treatment with dinaciclib significantly suppressed cell proliferation, induced caspase 3/7 levels and apoptotic activity in PDXC and CCA cell lines. Dinaciclib suppressed expression of its molecular targets CDK2/5/9, and anti-apoptotic BCL-XL and BCL2 proteins. Despite the presence of cyclin D1 amplification in the PDXC line, palbociclib treatment had no effect on cell proliferation, cell cycle or apoptosis in the PDXC as well as other CCA cell lines. Importantly, dinaciclib, in combination with gemcitabine, produced a robust and sustained inhibition of tumor progression in vivo in a PDX mouse model, greater than either of the treatments alone. Expression levels of two proliferative markers, phospho-histone H3 and Ki-67, were substantially suppressed in samples treated with the combination regimen. Our results identify dinaciclib as a novel and potent therapeutic agent alone or in combination with gemcitabine for the treatment of CCA.

## Introduction

Cholangiocarcinoma (CCA) is a highly invasive and rare malignancy of the biliary tree arising from epithelial cells. CCA typically presents at an advanced stage, and tumors are multifocal in nature. It is the second-most common primary hepatic cancer and accounts for 10–20% of all hepatobiliary malignancies^1^. CCA is non-responsive to therapy, with a poor prognosis and a survival rate of less than 5% over 5 years^2^. The global incidence and mortality of CCA is increasing rapidly. The standard of care for CCA treatment consists of the combination of gemcitabine and cisplatin, with no other effective treatment options existing for patients after progression on this regimen. Thus, a concerted effort is needed to develop novel therapies that work through different mechanisms compared to existing therapies.

Cyclin-dependent kinases (CDKs) are a well-characterized family of serine/threonine kinases that regulate cell cycle progression^3^. CDK-dependent inactivation of RB results in repression of multiple genes encoding proteins required for DNA synthesis (S phase) or mitosis^3^. CDKs also play important roles in neural development^4^ and transcription^5^. CDK4 and CDK6 phosphorylate transcriptional regulators such as RB or Smads^3,6^. The archetypal cell-cycle kinase CDK1 phosphorylates multiple transcription factors and epigenetic modulators^7^. By contrast, major transcriptional CDKs such as CDK7 or CDK11 directly control cell-cycle progression, in some instances independently of transcription. Disruption of cell cycle control and aberrant activation of CDKs is a hallmark of human cancers^8,9^. Due to the critical role of CDKs in regulating cell cycle processes, targeting them with small molecule inhibitors represents an attractive and rational therapeutic strategy.

Several CDK4/6 inhibitors (palbociclib, ribociclib, and abemaciclib) have been developed in the last decade. CDK4 and CDK6 are serine/threonine kinases whose activity is regulated by various mechanisms, positively by association with cyclin D (D1–D3) and negatively by binding to CDK inhibitors of the INK4 family^10^. Palbociclib (PD0332991) is a specific CDK4/6 inhibitor that arrests cell cycle progression in proliferating tumor cells, and tumors lacking *RB1* have been shown to be refractory to its treatment^11^. Due to its promising results in preclinical models and in clinical trials in different cancers^12,13^, the Food and Drug Administration (FDA) approved it in combination with anti-estrogen therapies for the treatment of hormone receptor-positive breast cancer^14,15^.

Dinaciclib (SCH727965) is a potent, selective small molecule inhibitor of CDKs inhibiting CDK1, CDK2, CDK5 and CDK9 at nano-molar concentrations^16^. Dinaciclib has been reported to be active against a broad range of human cancer cell lines^16^ and inhibits tumor growth in preclinical models. Dinaciclib has a favorable safety and pharmacokinetic profile in mice^16^.

In the present study, we investigated the effects of dinaciclib and palbociclib on a CCA PDXC and established CCA cell lines. Dinaciclib treatment induced apoptosis and caspase 3/7 activity, and suppressed CDK2/5/9 protein expression levels in PDXC and CCA cell lines. Dinaciclib suppressed tumor growth in vivo in a PDX mouse model and produced robust and sustained antitumor activity when combined with gemcitabine, a chemotherapeutic drug used in the treatment of CCA. In contrast, palbociclib had no effect on CCA cytotoxicity or apoptosis in culture and did not suppress tumor growth in vivo. Taken together, our study suggests dinaciclib as an effective therapeutic agent, alone or in combination with gemcitabine, for CCA treatment.

## Results

### PDX generation and high throughput drug screen

**Figure 1:**
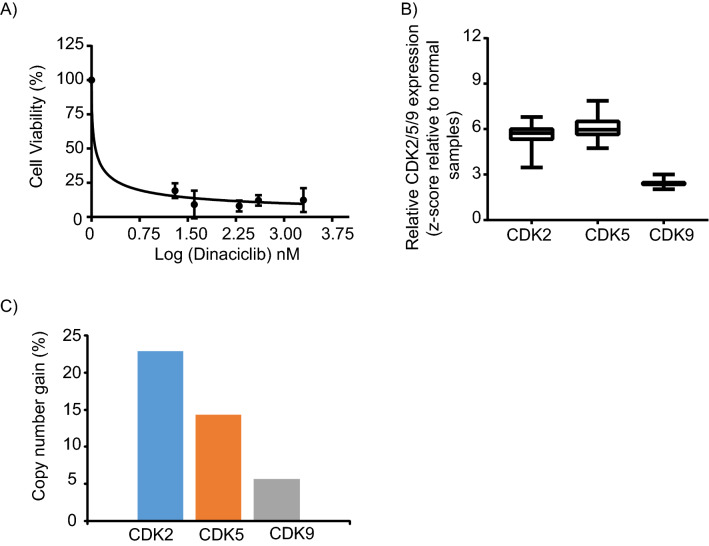
Dinaciclib treatment and expression of CDK2/5/9 in CCA. (**A**) CHNG6, patient derived xenograft cells (PDXC) were subjected to a high throughput-drug screening and is significantly sensitive to dinaciclib at low nanomolar concentrations. (**B**) Relative mRNA expression of CDK2/5/9 are overexpressed in cholangiocarcinoma samples relative to normal samples from cBioportal database. mRNA expression was available for thirty-five tumor and ten normal samples. Expression of CDK’s was normalized with normal samples and the data presented as box plots. (**C**) Copy number analysis of thirty-five tumor samples from the cBioportal database indicates subsets of CCA samples with copy number gain in CDK2, CDK5 and CDK9. Copy number gain is presented in percentages.

A patient derived xenograft (PDX) mouse model was successfully generated in NSG mice from a CCA patient tumor sample. Cells generated from the PDX model (referred to as PDXC), were grown in culture as spheroids under tumor stem cell conditions, without fetal bovine serum, to better conserve the phenotype and genotype of the original tumor, and to mimic a 3D tumor tissue environment^17^. We performed a high-throughput drug screen (HTDS)^18^ using the PDXC designated as CHNG6, and identified a high level of sensitivity to the CDK2/5/9 inhibitor, dinaciclib (Fig. 1A). As dinaciclib targets CDK2/5/9, we analyzed their mRNA expression levels and copy number in CCA samples from TCGA database employing the cBioportal site (cbioportal.org). Copy number and mRNA expression data were available for thirty-five tumor and ten normal samples. Relative CDK2/5/9 mRNA expression levels were overexpressed in CCA samples when compared to normal samples (Fig. 1B). The copy number analysis of CCA tumor samples indicated a gain in CDK2 (22.8%), CDK5 (14.3%), and CDK9 (5.7%) (Fig. 1C). Overexpression and copy number gain of CDK2/5/9 in CCA samples suggest their possible role in CCA progression and their potential utility as therapeutic targets. Since the HTDS identified sensitivity of CCA PDXC to dinaciclib, and CDK expression was elevated in CCA tumor samples, we assessed the therapeutic activity of dinaciclib in CCA.

### Dinaciclib suppresses cell growth and induces apoptosis

**Figure 2:**
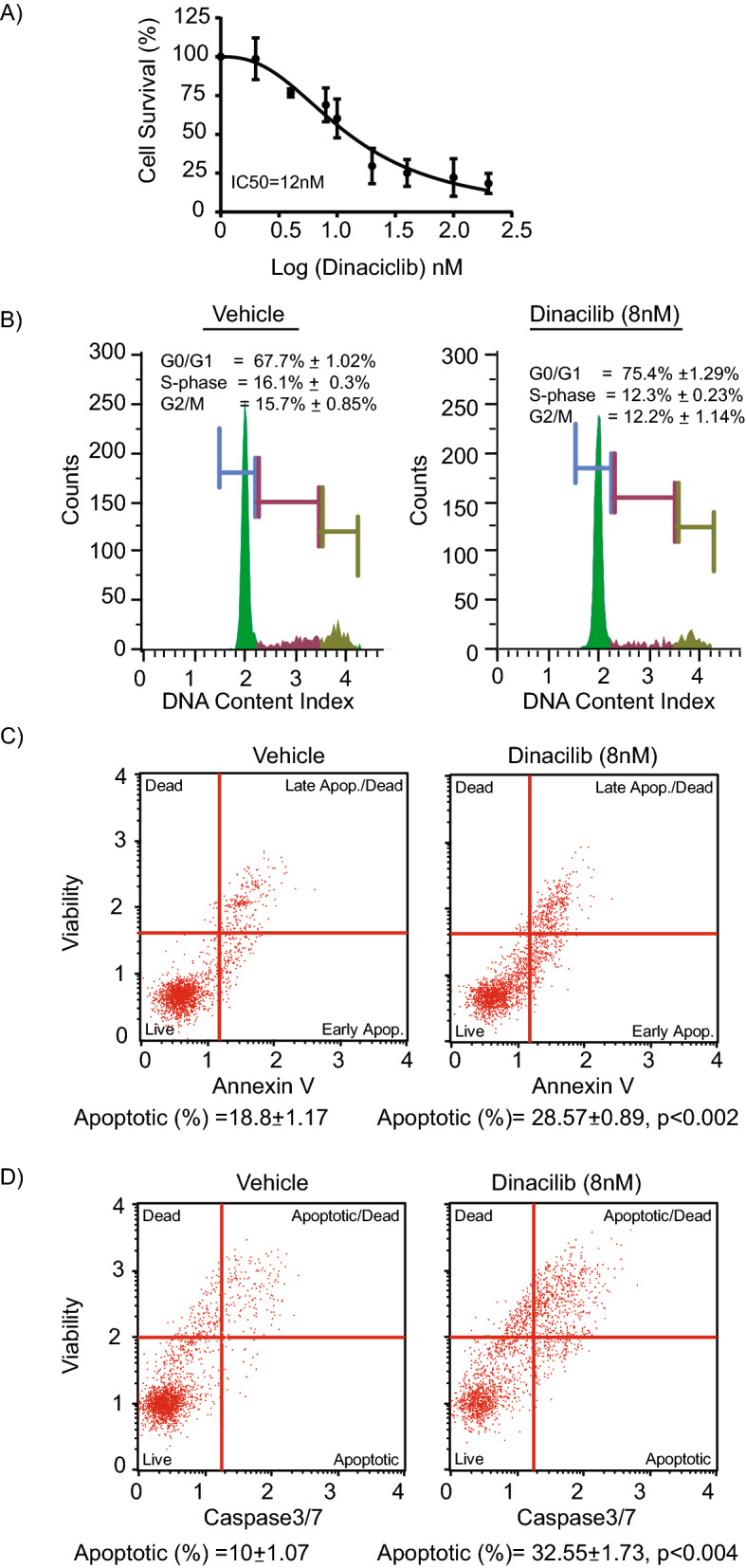
Effect of dinaciclib on patient derived xenograft cells. (**A**) Treatment of CHNG6 cells with dinaciclib for 24 h substantially reduced cell proliferative ability of these cells and the IC_50_ was in nanomolar range. (**B**) Cell cycle analysis indicated G0/G1 blockade and suppression in S-phase population with dinaciclib treatment (8 nM) for 24 h when compared to vehicle treatment. (**C**) Dinaciclib treatment (8 nM) of CHNG6 cells for 24 h substantially induced apoptosis when compared to vehicle treatment. (**D**) CHNG6 cells treated with dinaciclib (8 nM) for 24 h exhibited increased caspase 3/7 activity when compared to vehicle treatment.

Treatment with dinaciclib substantially suppressed the cell growth of CHNG6 cells, with an IC_50_ in the low nano-molar range (Fig. 2A). Dinaciclib treatment blocked the G0/G1 phase of the cell cycle, and resulted in reduced S-phase population in CHNG6 cells when compared to vehicle (DMSO) treatment (Fig. 2B). Dinaciclib treatment induced apoptosis in CHNG6 cells, accompanied by enhanced caspase3/7 activity when compared to vehicle treatment (Fig. 2C–D). These observations indicate the ability of dinaciclib to suppress the proliferative ability and enhance the apoptotic index in patient-derived xenograft CCA cells in culture.

### Palbociclib had no effect on CHNG6 cell survival or apoptosis

**Figure 3:**
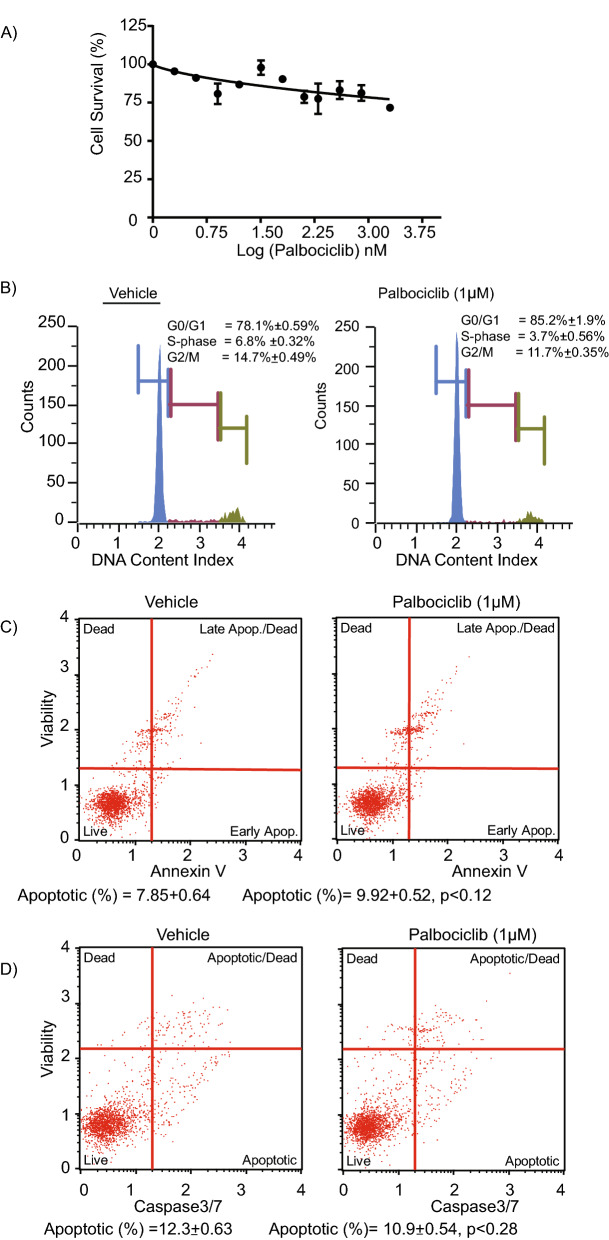
Effect of palbociclib on patient-derived xenograft cells. (**A**) Dose response curve of CHNG6 cells treated with palbociclib for 48 h had an insignificant effect on cell survival. (**B**) CHNG6 cells treated with palbociclib (1 µM) for 48 h induced modest G0/G1 arrest and reduction in S-phase population when compared to vehicle-treated cells. (**C**) Palbociclib treatment of CHNG6 for 48 h had no significant effect on induction of apoptosis when compared to vehicle-treated cells. (**D**) CHNG6 cells treated with palbociclib for 48 h had no effect on caspase 3/7 activity when compared to vehicle-treated cells.

Genomic analysis of the CCA patient sample from which the PDX was generated was performed by utilizing the CLIA-approved Foundation One CDx test from Foundation Medicine. CCND1, FGFR2 and FGFR3 were reported to be amplified in the patient sample. However, the tumor did not harbor mutations in the RB and TP53 genes. Due to the presence of CCND1 amplification, CHNG6 cells were also assessed for their sensitivity to palbociclib treatment. As palbociclib targets CDK4 and CDK6, their mRNA expression and copy number was analyzed employing TCGA database. mRNA expression levels of CDK4 and CDK6 were overexpressed in CCA tumor samples when compared to normal samples (Supplemental Fig. 1A). Copy number analysis indicated gain in CDK4 (25.7%) and CDK6 (14.7%) in CCA tumor samples (Supplemental Fig. 1B). The CDK4 and CDK6 overexpression and copy number gain in CCA tumor samples further suggest potential sensitivity of CCA to palbociclib treatment. However, palbociclib treatment had no impact on cell survival of CHNG6 cells, with a modest (10–15%) suppression of cell survival following palbociclib treatment (Fig. 3A). Palbociclib treatment caused minimal G0/G1 arrest and reduction in the S-phase population in CHNG6 cells (Fig. 3B). Palbociclib did not induce apoptosis or enhanced caspase3/7 activity in CHNG6 cells (Fig. 3C–D). Expression of CDK4 and CDK6 protein levels in CHNG6 cells were modestly suppressed by palbociclib treatment (Supplemental Fig. 1C). We further analyzed effects of palbociclib treatment on two established (KMCH and HuCCT1) CCA cell lines (Supplemental Fig. 2 and Supplemental Fig. 3). Palbociclib treatment had no effect on suppressing cell survival, inducing apoptosis or caspase3/7 activity in both cell lines. A G0/G1 block was observed in the HuCCT1 (Supplemental Fig. 2), but not in the KMCH cell line (Supplemental Fig. 3). Overall, these results showed that palbociclib treatment was ineffective at inhibiting cell survival or inducing programmed cell death in both PDXC and established CCA cell lines.

### Dinaciclib regulates cell survival and induces apoptosis in established CCA cells

**Figure 4:**
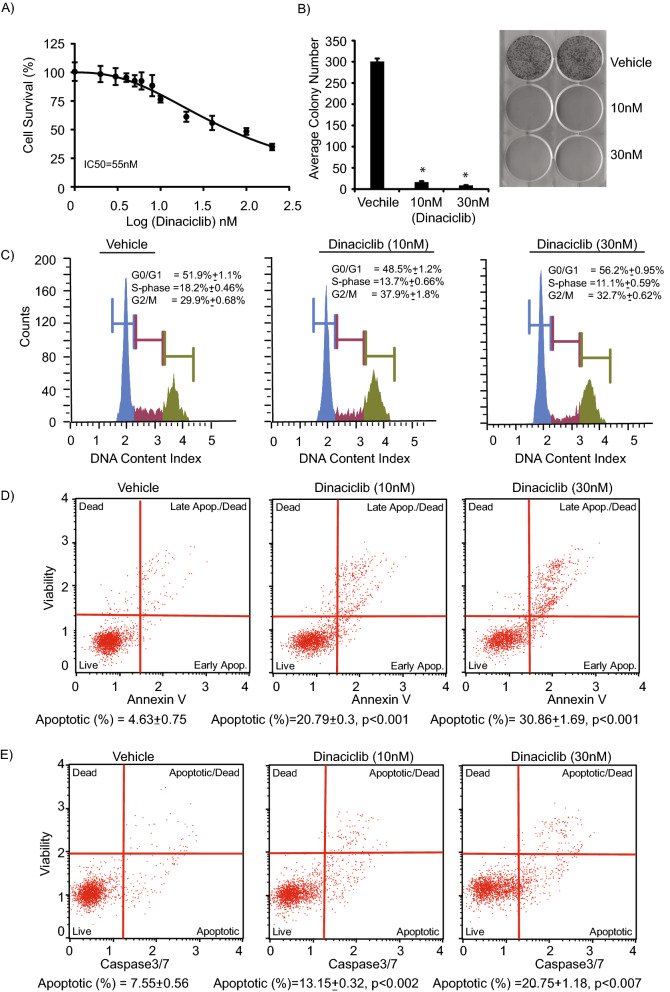
Effect of dinaciclib on HuCCT1 CCA cell line. (**A**) HuCCT1 cells treated with dinaciclib exhibited a substantial suppression in cell survival. (**B**) HuCCT1 cells treated with dinaciclib showed a substantial decrease in colony formation ability following dinaciclib treatment (10 and 30 nM). (**C**) Cell cycle analysis of HuCCT1 treated with dinaciclib (10 and 30 nM) for 24 h indicated suppression in S-phase population when compared to vehicle treatment. (**D**) Dinaciclib treatment of HuCCT1 cells (with increasing drug concentrations) for 24 h induced apoptosis. (**E**) Dinaciclib treatment of HuCCT1 cells enhanced caspase 3/7 activity when compared with vehicle treatment.

Next, to confirm dinaciclib’s anti-tumor effects, we employed two established CCA cell lines (HuCCT1 and KMCH). Dinaciclib treatment substantially suppressed cell survival and proliferation of HuCCT1 cells. Cell survival analysis indicated a high degree of sensitivity of HuCCT1 cells to dinaciclib treatment (Fig. 4A). A colony formation assay further confirmed the significant suppression in cell proliferation (Fig. 4B). Cell cycle analysis indicated a decrease in the S-phase population of the cell cycle, and a modest increase in the G2/M phase population, following dinaciclib treatment (Fig. 4C). A significant induction in the apoptotic population was observed following dinaciclib treatment in a dose-dependent manner (Fig. 4D). Furthermore, dinaciclib treatment increased caspase 3/7 activity in HuCCT1 cells when compared to vehicle treatment (Fig. 4E). To further confirm the effects of dinaciclib on established cell lines, another CCA cell line, KMCH, was treated with dinaciclib. As shown in Supplemental Fig. 4, dinaciclib treatment substantially suppressed the cell growth and proliferation of KMCH cells. A marked increase in the apoptotic population and caspase 3/7 activity was observed following dinaciclib treatment when compared to vehicle treatment.

### Dinaciclib inhibits CDK2/5/9 and anti-apoptotic gene expression

**Figure 5:**
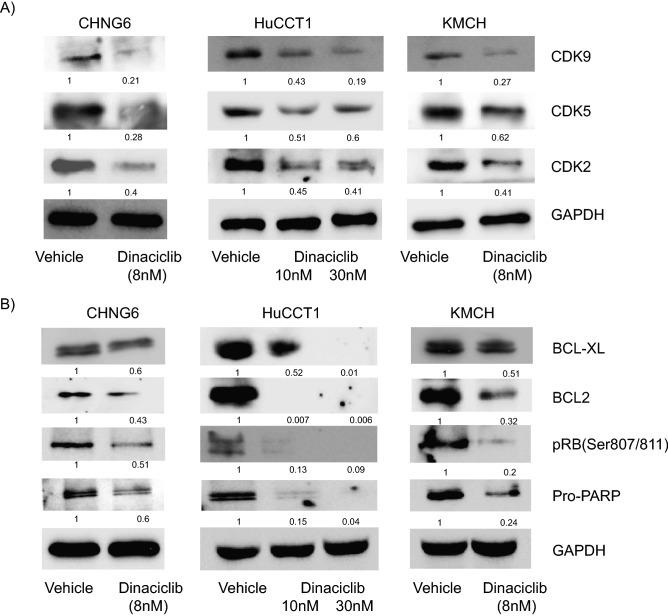
Effect of dinaciclib on its target genes. (**A**) Treatment of CHNG6 and established cell lines (HuCCT1 and KMCH) with varying doses of dinaciclib suppressed expression of CDK2, CDK5 and CDK9, the molecular targets of dinaciclib. (**B**) Dinaciclib treatment suppressed expression of anti-apoptotic genes BCL2 and BCL-XL and phosphorylation of RB in PDXC and two CCA cell lines. Quantification of western blot analysis was performed by Image J software and the numbers indicate the suppression in fold levels compared to vehicle treatment, denoted as 1. Original uncropped western blot pictures are provided in supplemental material.

Next, to understand the underlying mechanistic role of dinaciclib, we analyzed the effects of dinaciclib on its molecular targets (CDK2/5/9) and other pro-proliferative genes in CHNG6 and established CCA cell lines (KMCH and HuCCT1). Dinaciclib treatment in all cell lines suppressed the expression levels of its protein targets, CDK2/5/9 (Fig. 5A). Expression of BCL-XL and BCL2, members of the anti-apoptotic family, were substantially suppressed following dinaciclib treatment (Fig. 5B). Furthermore, dinaciclib enhanced cleavage of the PARP protein as indicated by the suppression in expression of pro-PARP (Fig. 5B).

### Dinaciclib in combination with gemcitabine suppressed in vivo CCA tumor growth

**Figure 6:**
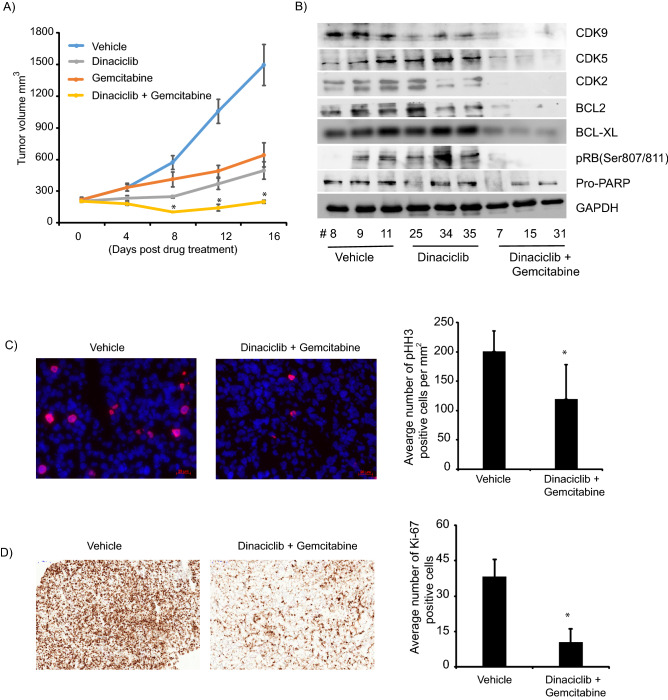
Effect of dinaciclib on in vivo tumor growth. (**A**) Dinaciclib in combination with gemcitabine significantly enhanced suppression of in vivo tumor growth than either of the treatments alone. (**B**) Western blot analysis of tumor samples from respective in vivo groups show reduction in expression of dinaciclib targets (CDK2/5/9) and anti-apoptotic proteins. Suppression in expression of these proteins was more pronounced in the combination treated group. Original uncropped western blot pictures are provided in supplemental material. (**C**) Representative immunofluorescence (IF) pictographs showing pHH3 positive stained cells (red) from vehicle and combination treated group. Bar graph showing average number of postitive pHH3 cells per mm^2^ of three samples each from vehicle and combination treated group. (**D**) Representaive immunohistochemistry (IHC) pictographs showing Ki-67 staining in vehicle and combination treated samples. Bar graph representing average number of Ki-67 positive cells of three samples each from vehicle and combination treated samples. Scale bar; IF = 20 µm and IHC = 100 µm. *p < 0.05.

Next, we explored the effects of dinaciclib alone and in combination with gemcitabine on in vivo CCA tumor growth. We performed an in vivo study in mice bearing tumors derived from CHNG6, as these tumors resemble the human tumor sample from which they were obtained. Dinaciclib and gemcitabine produced inhibition of tumor growth (Fig. 6A). In contrast, palbociclib treatment had no effect on in vivo tumor cell growth, which was consistent with results in culture (Supplemental Fig. 5). Dinaciclib in combination with gemcitabine produced a robust and sustained inhibition of tumor cell growth compared to either of the treatments alone (Fig. 6A). Protein was extracted from tumors from treated with vehicle (control), dinaciclib and the combination group for Western blot analysis. In the combination group, substantial suppression in the expression of dinaciclib targets (CDK2/5/9), as well as of BCL-XL and BCL2 was observed (Fig. 6B). Expression of phosphorylated histone H3 (Ser10), a mitotic marker, was significantly suppressed in in vivo samples from the combination treatment group when compared to vehicle samples, indicating suppression of tumor cell growth in dinaciclib/gemcitabine-treated samples (Fig. 6C). Furthermore, we analyzed expression of Ki-67, a proliferative marker, and observed it to be significantly suppressed in the combination treated group (Fig. 6D). pHH3 and Ki-67 expression was also anlayzed in the dinaciclib only treatment group. pHH3 expression was substantially suppressed, whereas, Ki-67 was modestly suppressed in the dinaciclib group when compared to the vehicle group (Supplemental Fig. 6).

## Discussion

Cholangiocarcinoma (CCA) is the most common neoplasm of the liver after hepatocellular carcinoma, and is responsible for 10–20% of hepatic malignancy deaths^19^. CCA patients present with unresectable or metastatic disease, with surgery being the only successful treatment modality. Despite systemic chemotherapy, prognosis remains poor and to date, there are no molecularly targeted therapies tailored to biliary tract cancer^20^, highlighting the importance of identifying new therapeutic options for CCA. In the present study, we provide evidence for the pre-clinical efficacy of dinaciclib in regulating CCA growth and apoptosis, employing a PDX model and established CCA cell lines.

Cell cycle deregulation is considered a hallmark of most tumors and is associated with activation of various CDKs^8,9^. CDKs are serine/threonine kinases that are involved in cell cycle progression and transcription in eukaryotic cells. Due to the functional significance of CDKs, they are becoming attractive and potent targets for anti-cancer therapies. Thus, developing highly selective and potent CDK inhibitors as novel therapeutic targets provide a strong rationale for the treatment of different cancers.

In this study, treatment of CCA PDXC with different antitumor agents in a HTDS revealed dinaciclib as the most active drug. Dinaciclib is a highly selective and potent CDK1/2/5/9 inhibitor^16^. Analysis of the TCGA database revealed mRNA overexpression and copy number gain of CDK2, 5 and 9 in CCA tumor samples. Specifically, copy number gain was present for CKD2 (22.8%), CDK5 (14.3%) and CDK9 (5.7%) in CCA specimens. Overexpression and copy number gain of dinaciclib targets, along with the sensitivity of CHNG6 to dinaciclib treatment, prompted us to pursue its efficacy as well as its mechanism of action in CCA. Dinaciclib is a second-generation CDK inhibitor, more potent and with a broader therapeutic index than flavopiridol, the first generation CDK inhibitor^21^. Flavopiridol is a well-studied pan-CDK inhibitor with few clinical benefits, partly due to high off-target effects and complex pharmacokinetics^22,23^. Other first generation CDK inhibitors, SNS-032^24^, (R)-roscovitine and PHA-793887^25^ have been discontinued in clinical studies, in part due to their lack of target specificity and potency. In contrast, dinaciclib is a highly potent and selective inhibitor of CDK1/2/5/9, with nanomolar anti-proliferative activity against different tumor types^16,26^. Dinaciclib treatment of CCA PDXC and established CCA cell lines significantly suppressed cell growth at low nanomolar concentrations. A significant reduction in the S-phase and induction in apoptosis was observed with dinaciclib treatment. The molecular targets of dinaciclib CDK2/5/9, along with pro-survival proteins BCL2 and BCL-XL, were substantially suppressed in cell lines following dinaciclib treatment. Dinaciclib-mediated inhibition of CDK9 has been reported to be effective in suppressing MYC-driven lymphomas by selectively targeting MCL-1, another BCL2 family member^27^. CCA samples overexpress MYC^28^ as well as CDK9, providing an additional potential rationale for their sensitivity to dinaciclib. BCL-XL expression levels are predictive biomarkers for dinaciclib-induced apoptosis and antitumor response in solid tumors^29^. We previously reported overexpression of BCL-XL in CCA^30^. Thus, detection of BCL-XL in CCA tumor samples may represent an additional strategy to identify CCA samples that may respond to dinaciclib. Dinaciclib, in combination with gemcitabine, significantly suppressed the in vivo tumor cell growth of CHNG6, and was more effective than either of the agents alone. This observation is in agreement with other studies, wherein synergistic anticancer effects were observed when dinaciclib was combined with other chemotherapeutic agents, including NVP-AUY92 and vinblastine in osteosarcoma and leukemia, respectively^31,32^. Rituximab, in combination with dinaciclib, was well tolerated in leukemia patients with favorable clinical activity^33^. Finally, lung cancer specimens with a *KRAS* mutation are more susceptible to dinaciclib treatment, as they undergo anaphase catastrophe^34^. CCA samples have a *KRAS* gain of function mutation in 15–25%^35^ of cases, suggesting that *KRAS*-mutant CCA samples may also be susceptible to dinaciclib treatment. Overall, these studies suggest that dinaciclib in combination with chemotherapeutics or targeted therapies could also be effective in the management of CCA.

Furthermore, our in vivo study showed substantial suppression in expression of proliferative markers pHH3 and Ki-67, confirming the potent anti-proliferative effects of the dinaciclib-gemcitabine drug combination. Elevated expression of pHH3 has been used as a marker of poor prognosis in different malignancies^36,37^. Our data suggest that the patient from which the PDX was derived would have benefitted from this drug regimen. However, based on current guidelines, the patient was treated with the cisplatin/gemcitabine combination after recurrence, which was poorly tolerated and had to be stopped due to toxicity. Our in vivo efficacy data suggest the combination of dinaciclib and gemcitabine as a novel therapeutic strategy for CCA patients. However, further studies need to be performed to fully comprehend the benefits of this combination on cholangiocarcinoma.

Molecular profiling analysis of the human tumor sample from which the PDX was derived revealed CCND1 amplification, suggesting its potential sensitivity to CDK4/6 inhibitors. Amplification and overexpression of CCND1 has been reported in many different tumors^38^. The Cyclin D-CDK4/6-INK4 pathway is frequently deregulated in various malignancies, and CDK4/6 have emerged as promising therapeutic targets. CDK4/6 amplification and overexpression is reported in breast and other tumors^39,40^. A recent study^41^ showed dependency of CCA on cyclin D-dependent kinase activity. The analysis of CCA tumor samples employing TCGA database indicated CDK4 and CDK6 overexpression versus normal samples. Similarly, analysis of the same dataset indicated copy number gain of CDK4 (25.7%) and CDK6 (14.7%) in CCA samples. These observations further suggested sensitivity of CCA to palbociclib treatment. We employed palbociclib, a FDA-approved CDK4/6 inhibitor, to treat the PDXC with cyclin D1 amplification. Intriguingly, palbociclib had no effect on viability, apoptosis or caspase 3/7 activity in the CCA PDXC. In addition, palbociclib had no effects on cell growth, proliferation or apoptosis in the established CCA cell lines tested. These findings suggest the limitations of palbociclib as a potential therapeutic agent for the treatment of CCA, even in the context of CCND1 amplification. Clearly, further investigations need to be pursued on a much larger sample set to establish the role of palbociclib or other CDK4/6 inhibitors in CCA.

In conclusion, this study highlights the overexpression and copy number elevations for various CDKs (including CDK2/5/9) in human CCA specimens. Furthermore, our data demonstrate, for the first time, the utility of dinaciclib, a well-tolerated cyclin-dependent kinase inhibitor, as a promising selective therapeutic option for treatment of CCA alone or in combination with gemcitabine.

## Methods

### Patient-derived xenograft (PDX) mouse model, cell culture and drugs

Patient sample acquisition was performed under an Institutional Review Board (IRB) protocol approved at California Pacific Medical Center in accordance with relevant guidelines and regulations. Informed consent was obtained from the patient (Male, 58yrs old, tumor moderately differentiated, and stage IV) in accordance with approved institutional guidelines. PDX generation, STR analysis and PDXC culture conditions are previously described by our group^30^. The human CCA HuCCT1 cell line was purchased from the Japanese Collection of Research Bioresources Cell Bank (JCRB, Japan) and KMCH was kindly provided by Dr. Gregory Gores (Mayo Clinic, MN). HuCCT1 and KMCH were grown in RPMI (Thermofisher Scientific, South San Francisco, CA) with 5% fetal bovine serum (JR Scientific, Woodland, CA) and 1 × penicillin/streptomycin (Thermofisher Scientific) at 37 °C in a 5% CO_2_ incubator. Cell lines tested negative for mycoplasma contamination using MycoFluor Mycoplasma Detection Kit (Thermofisher Scientific) following manufacturer’s instructions. Dinaciclib, gemcitabine and palbociclib were purchased from Selleck chemicals (Houston, TX).

### TCGA dataset for CDKs

mRNA expression level and copy number of CDK2, CDK4, CDK5, CDK6 and CDK9 of CCA and normal samples were obtained from the cBioportal database (cbioportal.org). mRNA expression and copy number data were available for thirty-five tumor and ten normal samples. For mRNA expression level of CDK’s, the z-score (expression of tumor sample-average expression of normal samples/s.d. of normal samples) of each tumor sample was calculated and data presented as a box plot. Copy number gain of CDK’s was available for thirty-five CCA samples. The data for copy number gain is presented as percentage values (samples with gain in copy number/total number of samples*100) in a bar graph.

### Colony formation assay

The colony formation assay was performed as previously described^30^. CCA cells (300–500) were plated in each well of a 6-well plate and treated next day with the respective drugs. Cells were allowed to grow till visible colonies appeared usually 6–10 days after treatment. Colonies were stained with crystal violet (Sigma-Aldrich, St. Louis, MO) and counted. Data is presented as a bar graph of average number of colonies along with the pictograph of colonies.

### Cell viability assay

Cell viability was assessed as described^30^. Briefly, CCA cells (1000–2000) were plated in a 96 well plate and treated next day with either dinaciclib or palbociclib. Cell viability was assessed by using the Cell Counting Kit-8 (Dojindo Molecular Technologies, Rockville, MD) after 24 h of drug treatment following manufacturer’s instructions. 20µL of Cell Counting Kit-8 was added to each well and the plate was incubated at 37 °C for 2 h. Absorbance was read at 450 nm.

## Immunofluorescence and Immunohistochemistry

Immunofluorescence (IF) was performed as described earlier^42^. The anti-phospho histone H3 (Ser10) (1:500, Cell signaling) was detected using a secondary antibody labeled with Alexa Fluor 594 (1:1000, Molecular Probes). Three samples from vehicle and treated group were processed for IF. Mosaic images were acquired with 20 × magnification at a fixed exposure with a Zeiss Axio Imager Z2 microscope controlled by Axio Vision software. pHH3 positive cells were counted using ImageJ software and reported as count of positive cells per area (mm^2^).

Immunhistochemistry (IHC) analysis was performed as described earlier^43^. IHC for Ki-67 was performed by employing Ventana Benchmark autostainer (Ventana Medical Systems, Tucson, AZ) using CONFIRM anti-Ki-67 (30-9) antibody (Ventana Medical Systems). Ki-67 positive stained cells were identified by the presence of brown nuclear staining in tumor cells. Ki-67 positive stained cells were counted from 10 randomly unbiased selected areas from each vehicle and treated sample. Three samples from vehicle and treated groups were stained. Ki-67 positive stained cells are reported as the average number of counts from 10 areas.

### Cell cycle, annexin V and caspase 3/7 assays

Cell cycle, Annexin V and caspase 3/7 assays were performed by using the Muse cell cycle kit, Muse Annexin V apoptosis kit, and Muse Caspase 3/7 kit, respectively (EMD Millipore, Hayward, CA) following the manufacturer’s instructions. Cells were plated and treated on the next day with the respective drugs for 24 h or 48 h for these studies. For the cell cycle assay, cells were trypsinized, washed with PBS, fixed with cold ethanol and incubated at − 20 °C for 2 h. Cells were centrifuged, washed with PBS, followed by addition of 200µL of cell cycle reagent. Cells were incubated for 30 min in the dark at room temperature and analyzed by Muse Cell Analyzer. For the apoptosis assay, cells were trypsinized, and washed with PBS. Cells were resuspended in 100µL of apoptosis reagent and 100µL of 5% RPMI-1640 media and incubated for 20 min at room temperature and analyzed by Muse Cell Anlayzer. For the caspase 3/7 assay, cells were trypsinized, washed with PBS and resuspended in 50µL of assay buffer containing caspase 3/7 reagent and incubated at 37 °C for 30 min. 150µL of assay buffer containing 7-AAD was added and the cells analyzed by Muse Cell analyzer. Data for all assays were analyzed by Muse Cell Analyzer software.

### Western blot analysis

Cells were treated with respective drugs for 48 h. Protein was extracted from treated cells using RIPA buffer containing 1 × Halt protease inhibitor cocktail and 1 × Halt phosphatase inhibitor cocktail (Pierce, Rockford, IL). Proteins (10–25µg) from drug treated samples were subjected to SDS/polyacrylamide gel electrophoresis (PAGE) and transferred onto a nitrocellulose membrane. Nitrocellulose membranes were cut and hybridized with different antibodies. In some cases, proteins from two drug treated cell lines were run onto the same gel and hybridized with the same antibody. Specific antibodies against BCL2 #4223, BCL-XL #2764, CDK4 #12790, CDK6#13331, PARP #9532, CDK5 #2506 (Cell Signaling Technology, Danvers, MA), CDK9 #A303-493A, CDK2 #A301-812A, (Bethyl Laboratories, Montgomery, TX) and GAPDH #MAB374 (EMD Millipore, Hayward CA) were used. BioRad Chemidoc instrument (BioRad, Hercules, CA) was employed to capture and process the images of higher magnification. Original western blot pictures with low and high exposures wherever applicable are provided in the supplemental material.

### In vivo study

Six-week old NOD scid gamma (NSG) mice were purchased from Jackson Laboratories, Sacramento, CA. Mice where housed in a single ventilated cage in groups of four, provided environmental enrichment materials, and free access to water and food. Mice were maintained in a parasite and virus free animal facility under a 10-h dark and 14-h light cycle. In vivo studies were carried out in accordance with the National Institutes of Health guidelines, Health Research Extension Act of 1985 and the Public Health Service Policy on Humane Care and Use of Laboratory Animals (Policy), Office of Laboratory Animal Welfare assurance, and an approved Institutional Animal Care and Use Committee (IACUC) protocol. CHNG6 cells (0.5 × 10^6^) were mixed with 50% matrigel for subcutaneous injections in a total volume of 100 µl in the mouse flank. Once tumors were palpable, mice were randomized and divided into groups with average tumor volumes of 200mm^3^. Mice were divided into different treatment groups, vehicle (n = 6), dinaciclib (n = 8), palbociclib (n = 6), gemcitabine (n = 6) and dinaciclib plus gemcitabine combination group (n = 10). All drugs were administered intraperitoneally (i.p.) for 16 days. Drugs were diluted in 4% DMSO, 4% Tween-80 and 92% saline. Dinaciclib (20 mg/kg), palbociclib (25 mg/kg) were administered thrice weekly, whereas gemcitabine (15 mg/kg) was administered twice weekly. Toxicity studies were performed to determine the optimal tolerable dose for single and combination drugs. Mice treated with gemcitabine and combination drugs were on supportive care (nutritional and hydration gels) during the entire study. No toxicities were observed in mice treated with single agents; two mice in the combination group lost weight towards the end of the study. Tumors were measured by caliper and volumes were calculated as a product of (length x width x width)/2. Mice were sacrificed and tumors were collected and processed for immunofluorescence, immunohistochemistry and protein extraction.

### Statistical analysis

Statistical analyses were performed using GraphPad Prism 7 software (San Diego, CA). Differences in tumor growth between treatment groups were evaluated using two-way ANOVA repeated measures, and a Tukey’s multiple comparisons test. Statistical significance was defined as a *p* value < 0.05. Image J software was used to analyze western blot intensities.

## Supplementary information

Supplementary Information
