# Supplementary material for: Dinaciclib, a cyclin-dependent kinase inhibitor, suppresses cholangiocarcinoma growth by targeting CDK2/5/9
Source: Sci Rep. 2020 Oct 28;10:18489. doi: 10.1038/s41598-020-75578-5 (PMC7595101; doi:10.1038/s41598-020-75578-5)

Dinaciclib, a Cyclin-Dependent Kinase Inhibitor, Suppresses Cholangiocarcinoma Growth by Targeting CDK2/5/9

Hera Saqub[1], Hannah Proetsch-Gugerbauer[1], Vladimir Bezrookove[1], Mehdi Nosrati[1], Edith M Vaquero[1], David de Semir[1], Ryan J Ice[1], Sean McAllister[1], Liliana Soroceanu[1], Mohammed Kashani-Sabet[1], Robert Osorio[1] and Altaf A Dar[1]*

[1]California Pacific Medical Center Research Institute, 475 Brannan St, Suite 130, San Francisco, CA 94107 USA.

Supplemental Figure Legends

Supplemental Figure 1: CDK4 and CDK6 expression and copy number in CCA. A) Relative mRNA expression of CDK4 and CDK6 are overexpressed in cholangiocarcinoma samples relative to normal samples from cBioportal database. mRNA expression was available for thirty-five tumor and ten normal samples. B) Copy number analysis of thirty-five tumor samples from the cBioportal database indicates subsets of CCA samples with copy number gain in CDK4 and CDK6. Copy number gain is presented in percentages. C) Western blot analysis indicates modest suppression in CDK4 and CDK6 protein levels following palbociclib treatment of CHNG6 cells.

Supplemental Figure 2: Effects of palbociclib on HuCCT1 cells. A) Palbociclib treatment for 48hr did not suppress cell survival of HuCCT1 cells. B) Palbociclib treatment had no significant effect on reducing colony formation ability of HuCCT1 cells. C) Cell cycle analysis of HuCCT1 with palbociclib treatment induced arrest of G0/G1 cell population. D) HuCCT1 cells treated with palbociclib had modest effects on induction of apoptosis. E) Palbociclib treatment of HuCCT1 cells had no effect on enhancing caspase3/7 activity.

Supplemental Figure 3: Effects of palbociclib on KMCH cells. A) Palbociclib treatment had no effect on cell survival of KMCH cells. B) Palbociclib treatment did not suppress the colony formation ability of KMCH cells. C) Cell cycle analysis of KMCH cells treated with palbociclib had no effect on S-phase cell population. D) KMCH cells treated with palbociclib showed no significant effect on induction of apoptosis. E) KMCH cells treated with palbociclib exhibited no increase in caspase3/7 activity.

Supplemental Figure 4: Effect of dinaciclib on the KMCH CCA cell line. A) Cell survival analysis indicated a significant suppression in KMCH cells with dinaciclib treatment. B) Dinaciclib treatment substantially suppressed colony formation ability of KMCH cells. C) Cell cycle analysis indicated suppression in S-phase cell population with dinaciclib treatment. D) Dinaciclib treatment of KMCH cells substantially induced apoptosis when compared to vehicle treatment. E) KMCH cells treated with dinaciclib exhibited significant increase in caspase3/7 activity.

Supplemental Figure 5: Effect of palbociclib on *in vivo* tumor growth. A) Palbociclib treatment had no effect on reducing *in vivo* tumor growth.

Supplemental Figure 6: A) Representative immunofluorescence (IF) pictographs showing pHH3 positive stained cells (red) from vehicle and dinaciclib treated group. Bar graph showing average number of postitive pHH3 cells per mm^2 of three samples each from vehicle and dinaciclib treated group. B) Representaive immunohistochemistry (IHC) pictographs showing Ki-67 staining in vehicle and dinaciclib treated samples. Bar graph representing average number of Ki-67 positive cells of three samples each from vehicle and dinaciclib treated samples. Scale bar; IF=20µm and IHC=100µm. *$p<0.05$

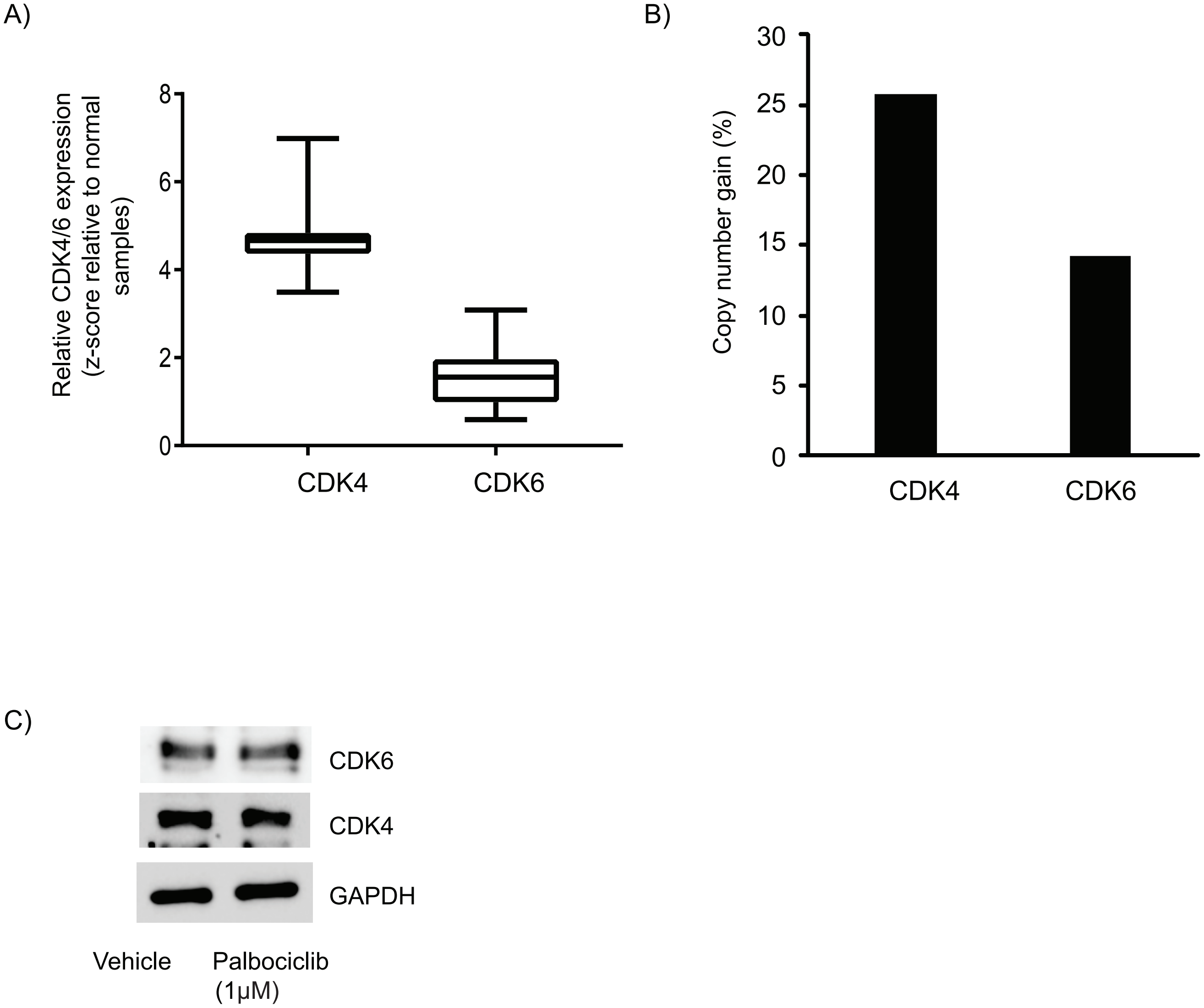

Supplemental Figure 1

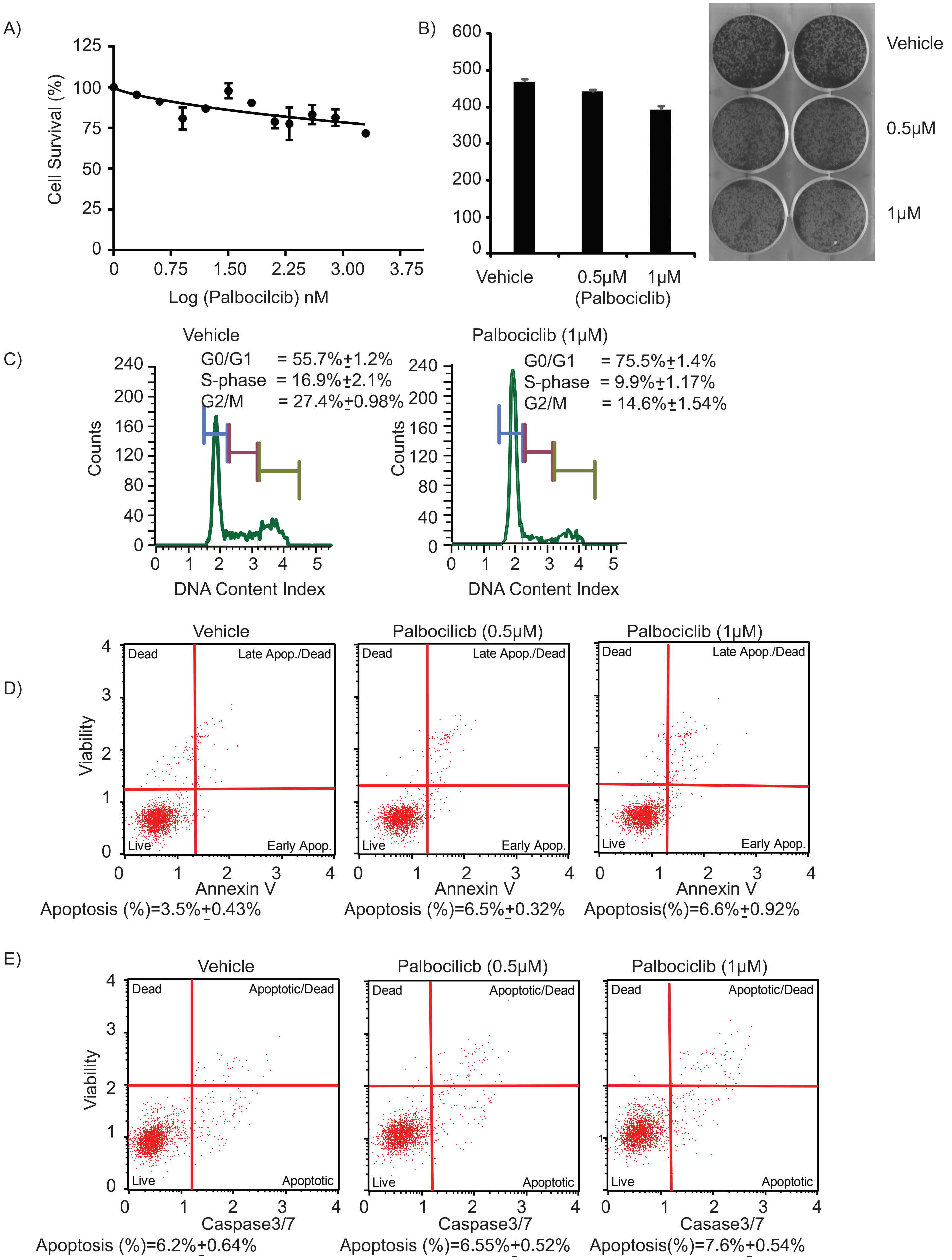

Supplemental Figure 2

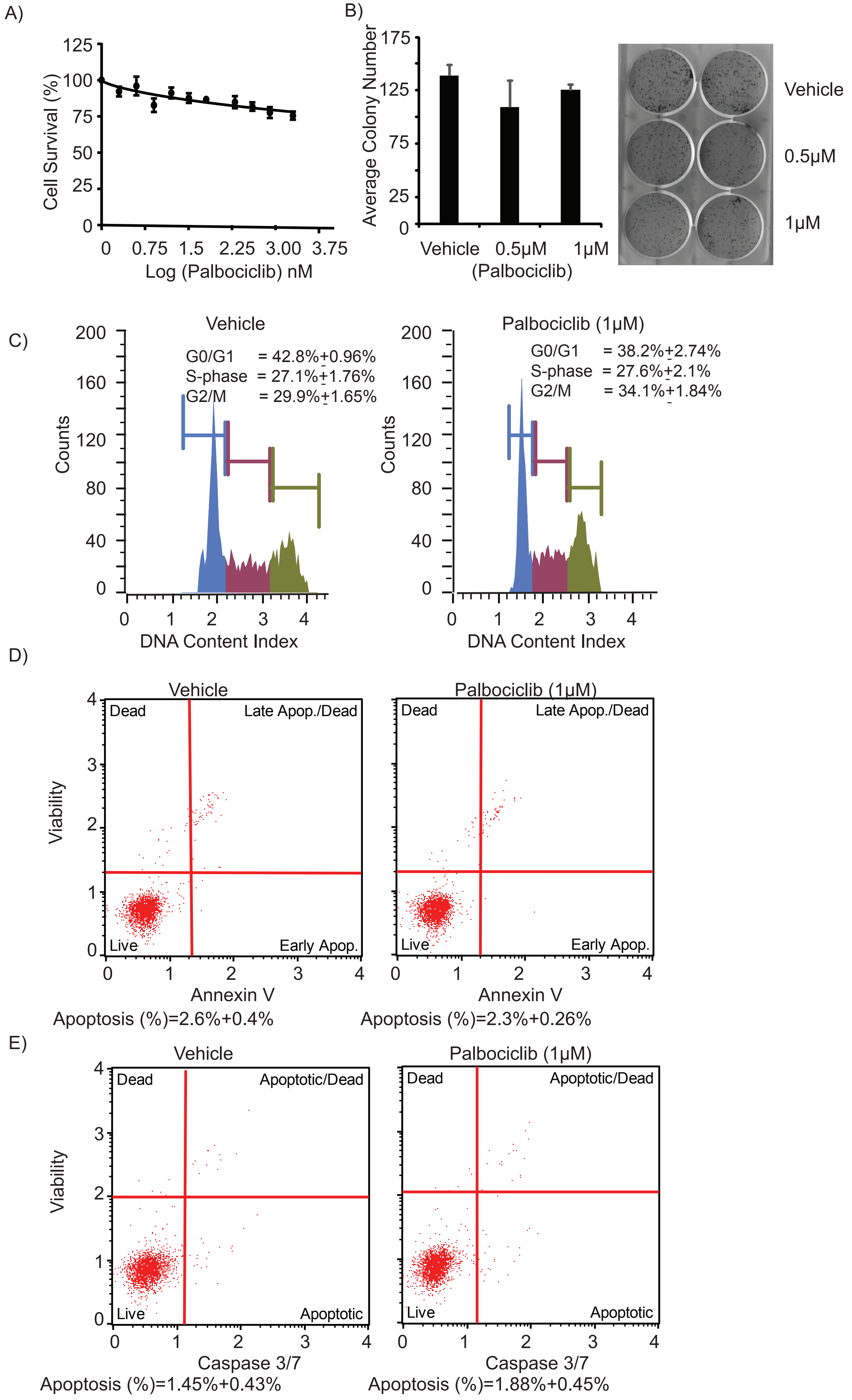

Supplemental Figure 3

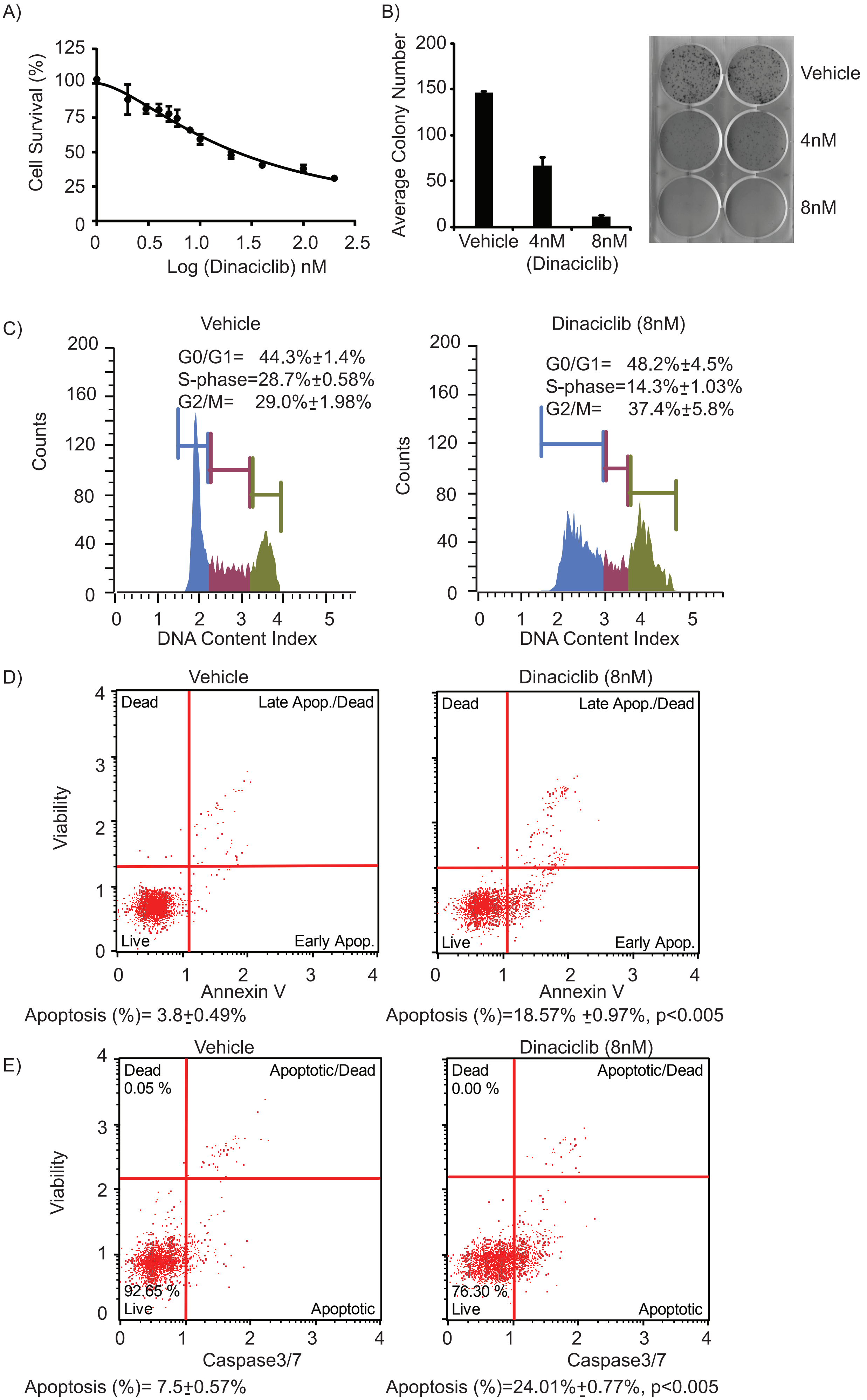

Supplemental Figure 4

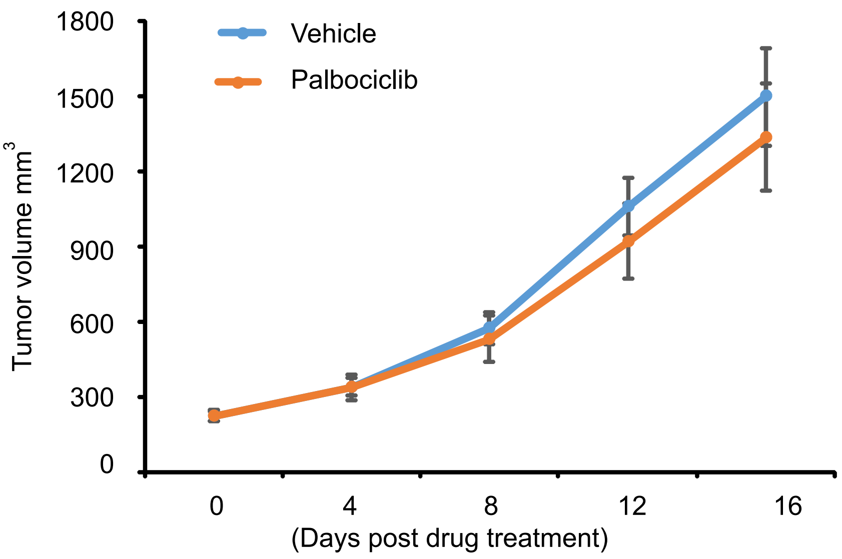

Supplemental Figure 5

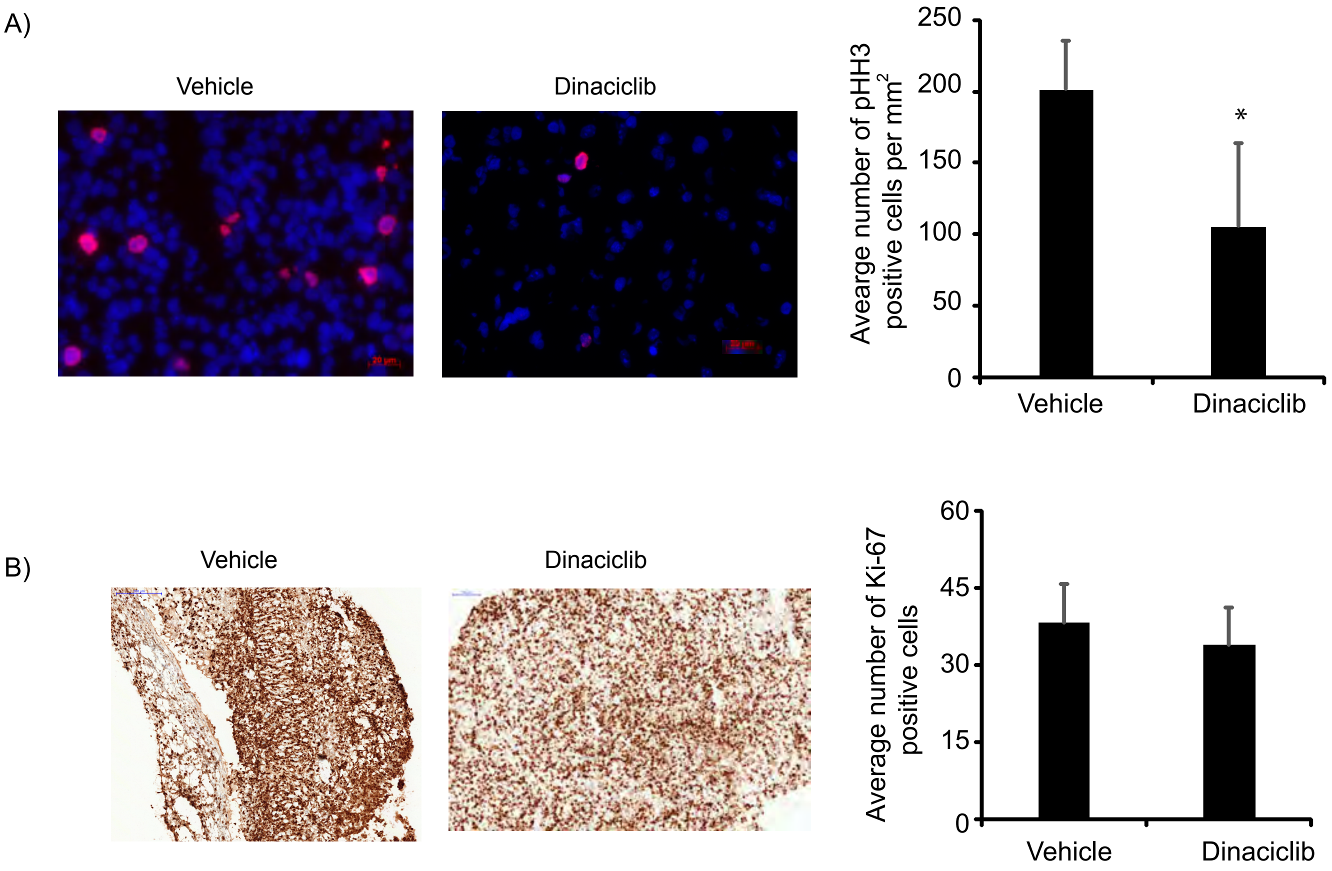

Supplemental Figure 6

Original Western blots for Figure 5A and 5B

Fig 5A CHNG6 CDK9

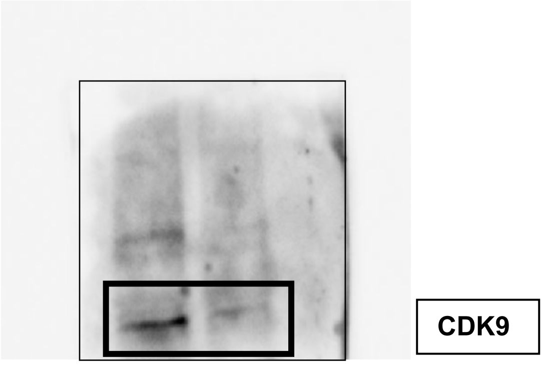

Fig 5A CHNG6 CDK2 (last two lanes)

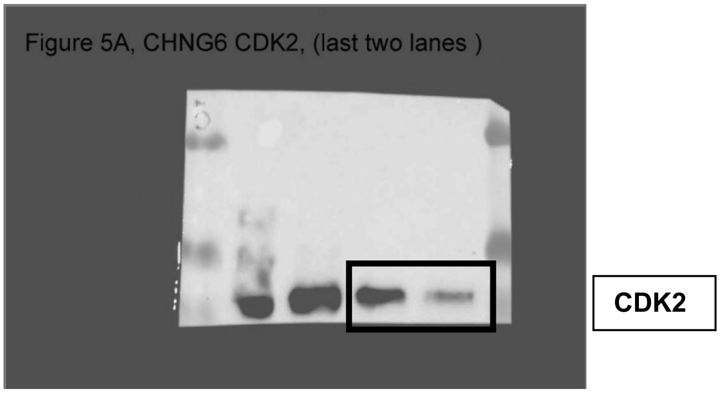

Fig 5A CHNG6 CDK5 (High exposure)

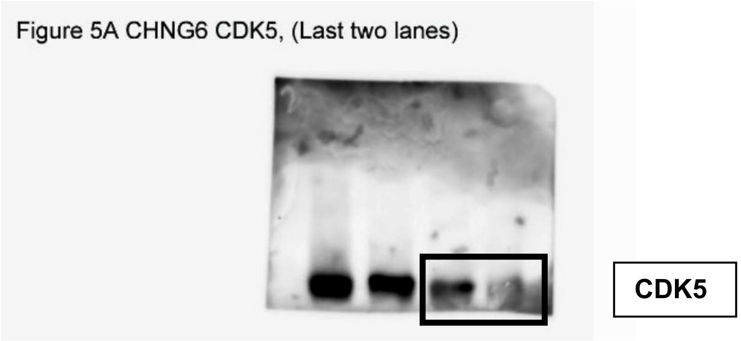

Fig 5A CHNG6 CDK5 (Low exposure)

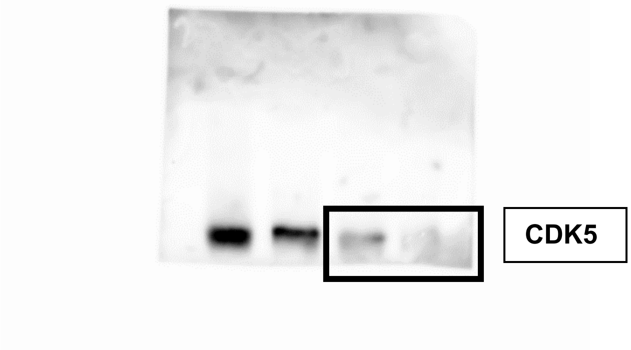

Fig 5A CHNG6 GAPDH (High exposure)

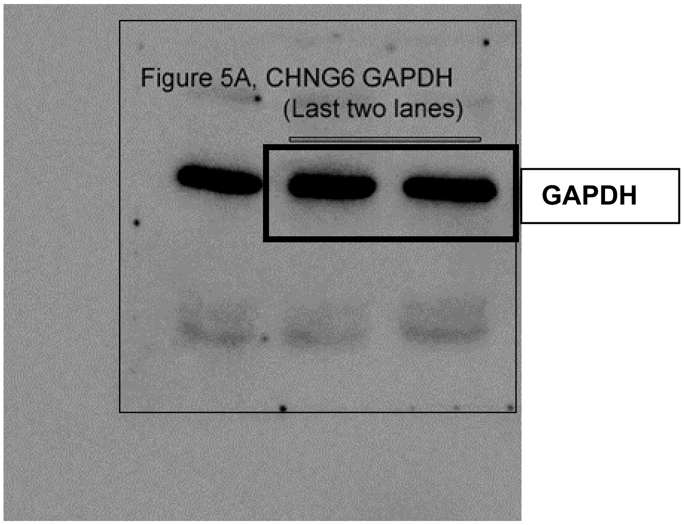

Fig 5A CHNG6 GAPDH (Low exposure)

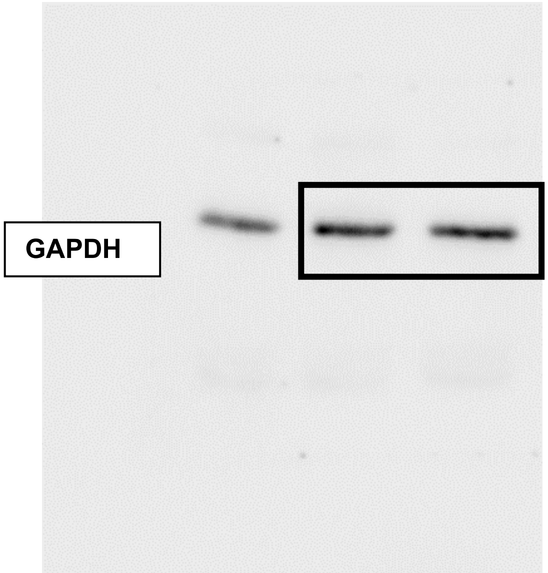

(thin lined black rectangles represent blot size).

Fig 5a HuCCT1 CDK9 (lower band)

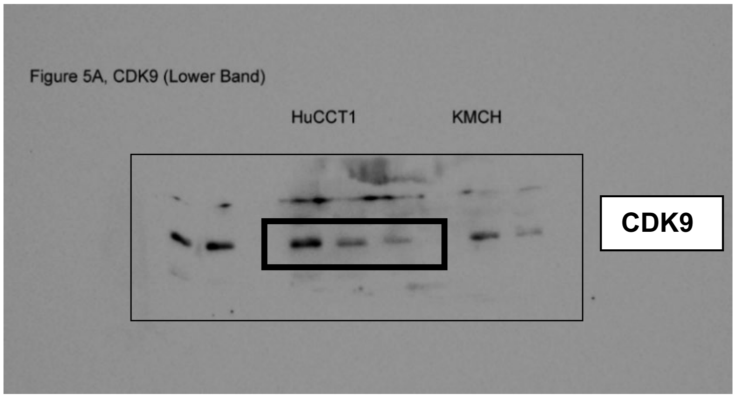

Fig 5a HuCCT1 CDK5 (high exposure)

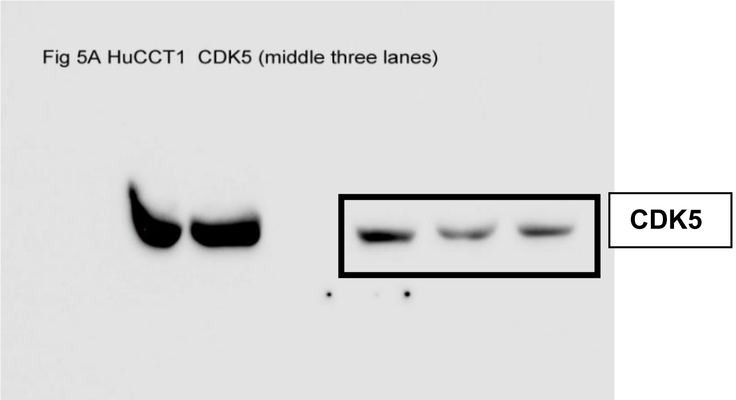

Fig 5a HuCCT1 CDK5 (Low exposure)

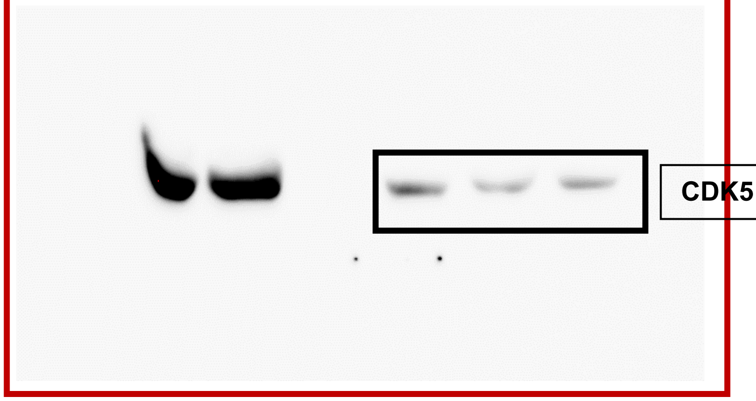

Fig 5a HuCCT1 CDK2 (High exposure)

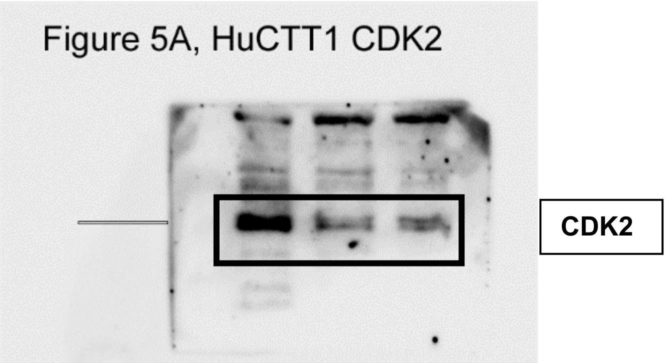

Fig 5a HuCCT1 CDK2 (Low exposure)

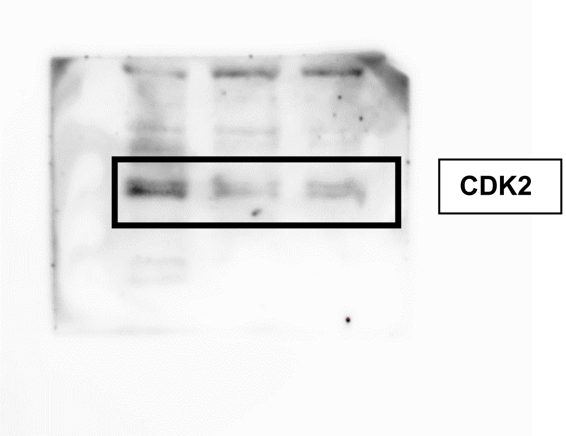

Fig 5a HuCCT1 GAPDH

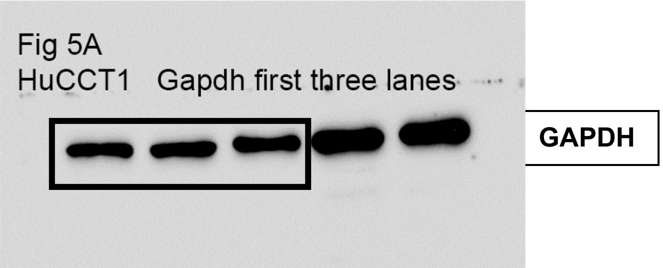

Fig 5a KMCH CDK9

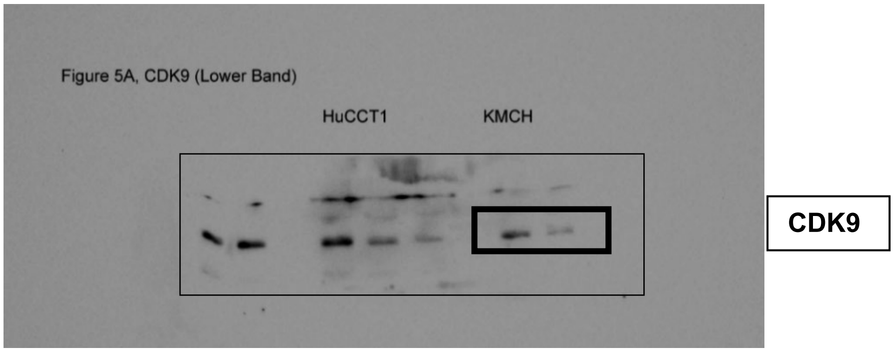

Fig 5a KMCH CDK5 (High exposure)

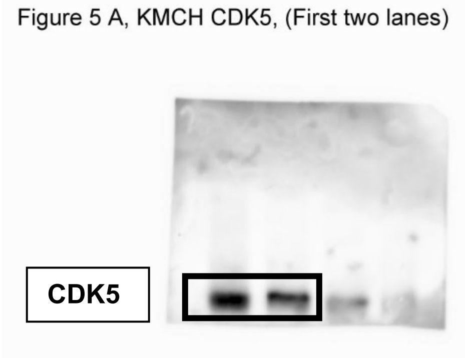

Fig 5a KMCH CDK5 (Low exposure)

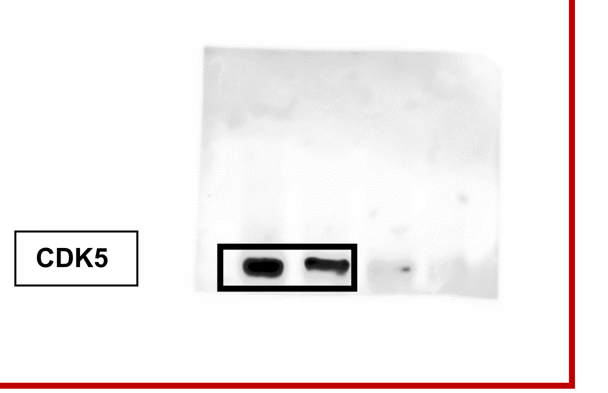

Fig 5a KMCH CDK2

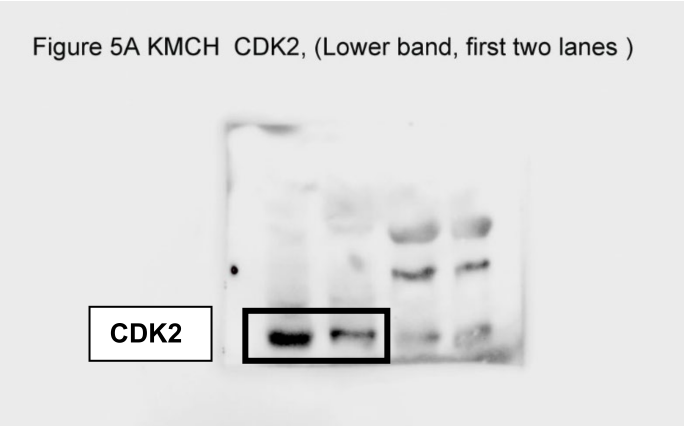

Fig 5a KMCH GAPDH

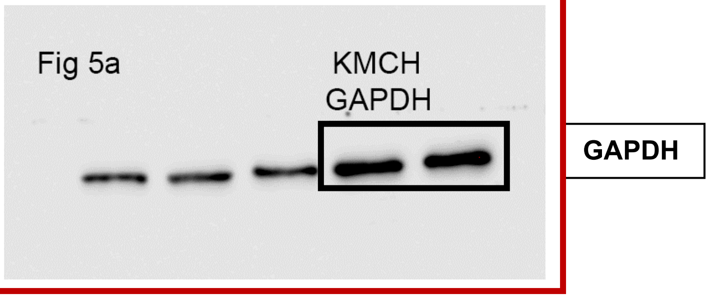

(thin lined black rectangles represent blot size).

Fig 5b CHNG6 BCL-Xl: Low exposure

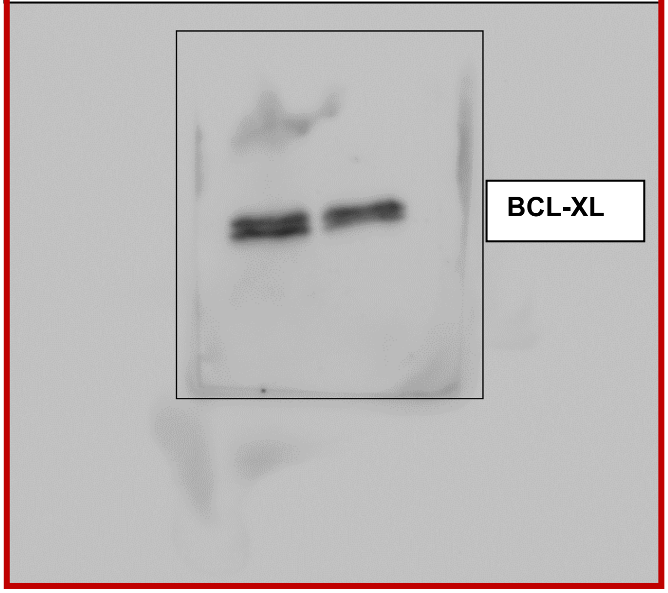

Fig 5b CHNG6 BCL2

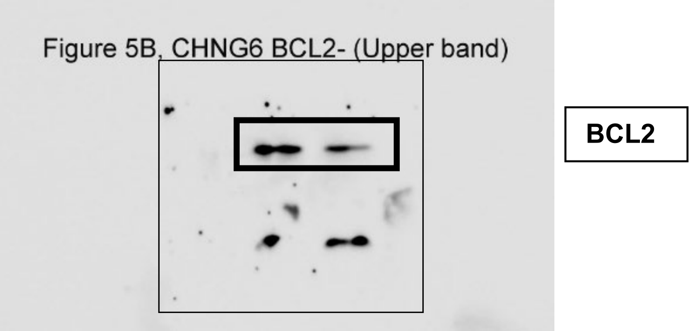

Fig 5b CHNG6 pRB

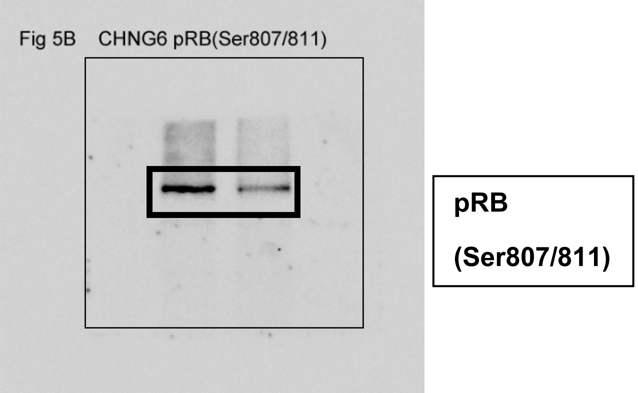

Fig 5b CHNG6 GAPDH

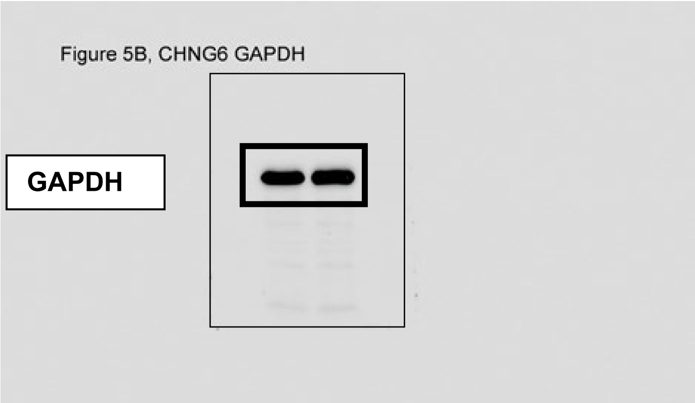

Fig 5b CHNG6 pro-PARP (High exposure)

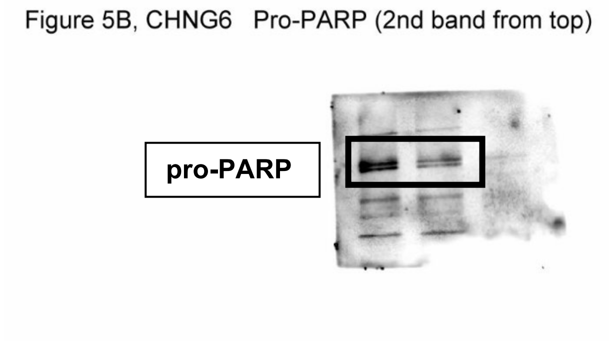

Fig 5b CHNG6 pro-PARP (Low exposure)

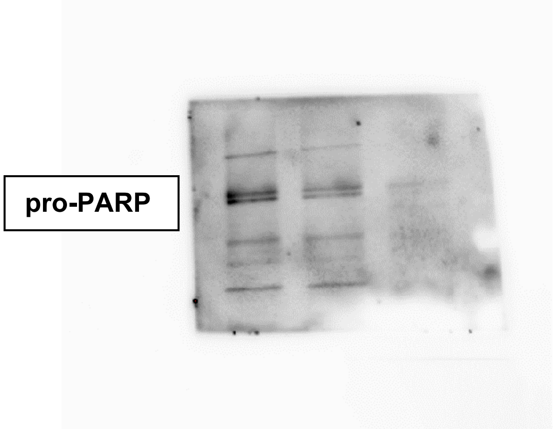

(thin lined black rectangles represent blots.

Fig 5b HuCCT1 BCL-XL

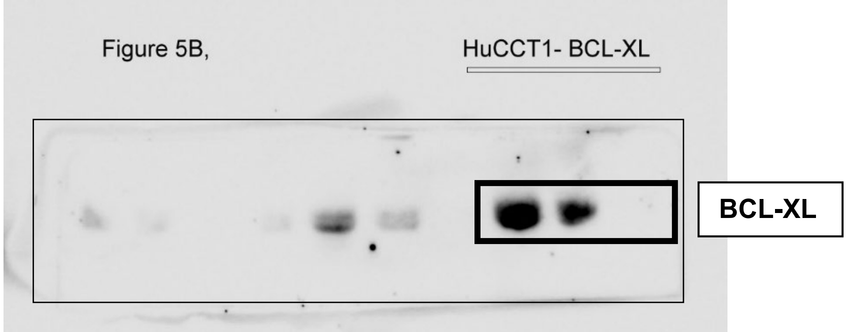

Fig 5b HuCCT1 pRB

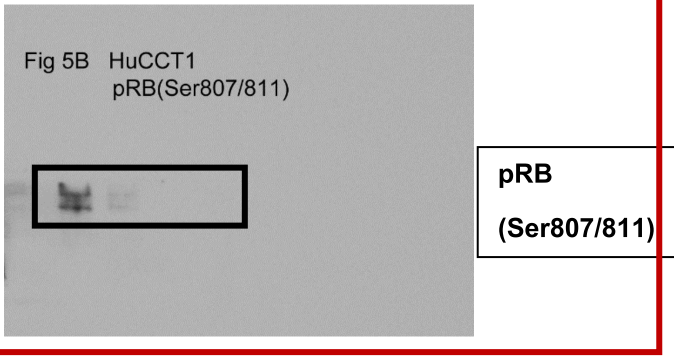

Fig 5b HuCCT1 BCL2 (High exposure)

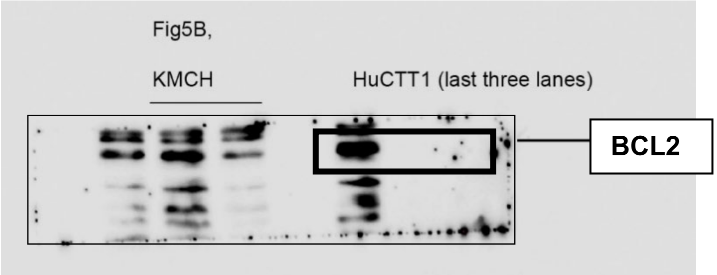

Fig 5b HuCCT1 BCL2 (Low exposure)

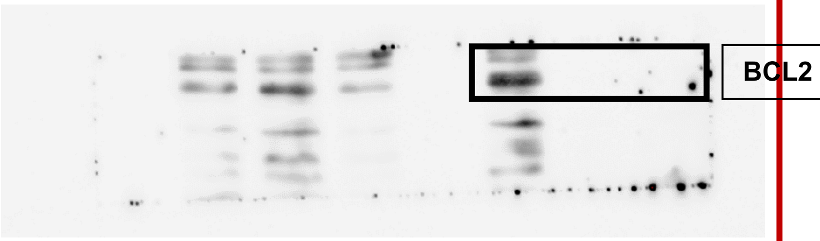

Fig 5b HuCCT1 pro-PARP (High exposure)

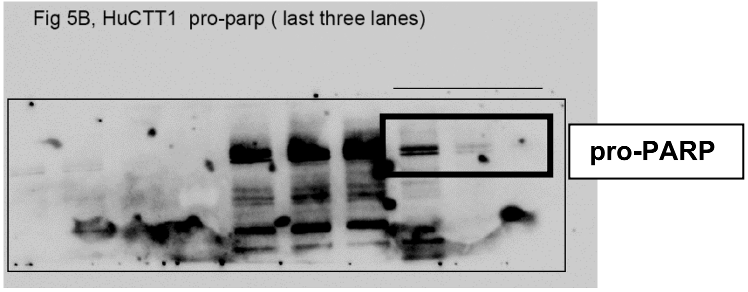

Fig 5b HuCCT1 pro-PARP (Low exposure)

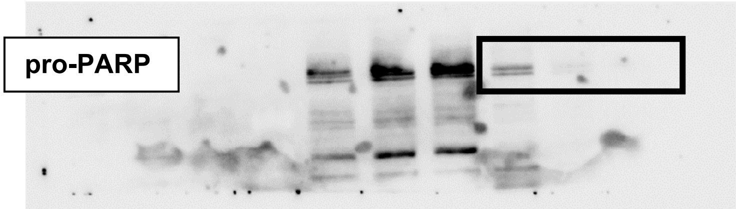

Fig 5b HuCCT1 GAPDH (High exposure)

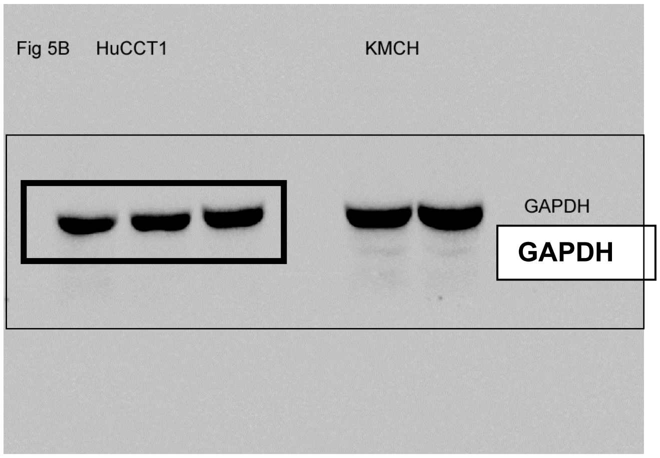

Fig 5b HuCCT1 GAPDH (Low exposure)

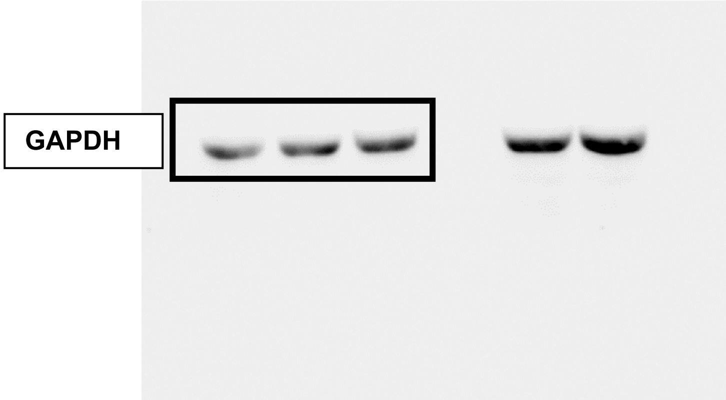

(thin lined black rectangles represent blot size).

Fig 5b KMCH BCL-XL: Low exposure

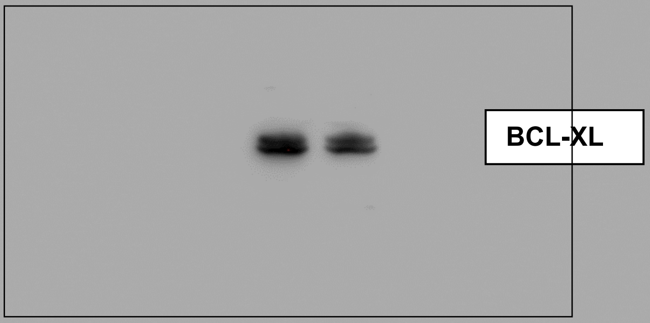

Fig 5b KMCH BCL-XL: High exposure

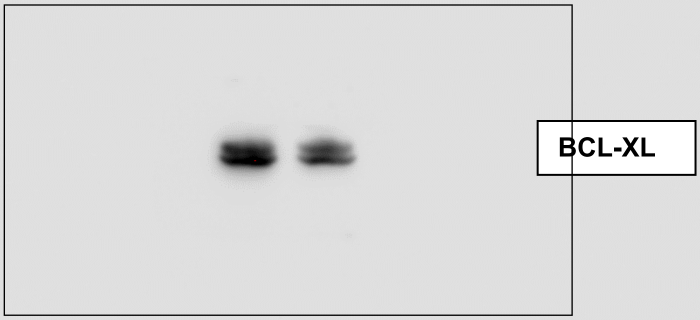

Fig 5b KMCH pRB

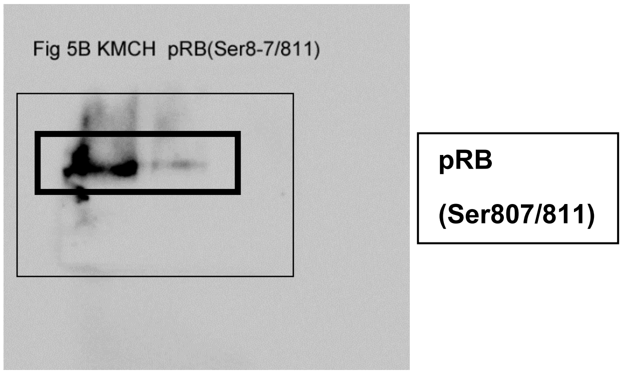

Fig 5b KMCH BCL2 (High exposure)

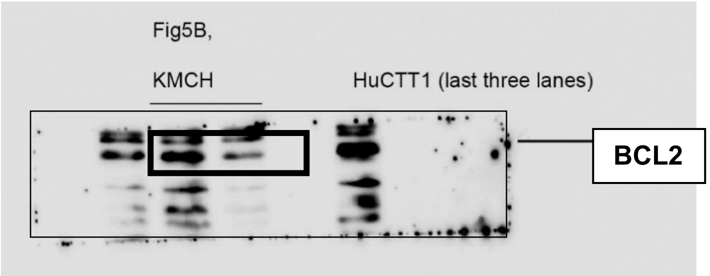

Fig 5b KMCH BCL2 (Low exposure)

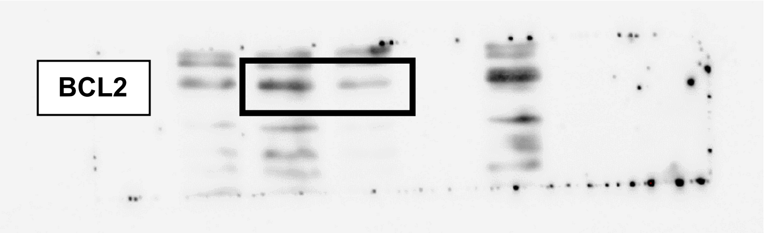

Fig 5b KMCH pro-PARP

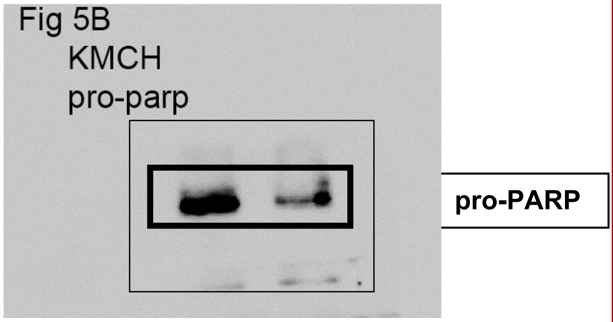

Fig 5b KMCH GAPDH (High exposure)

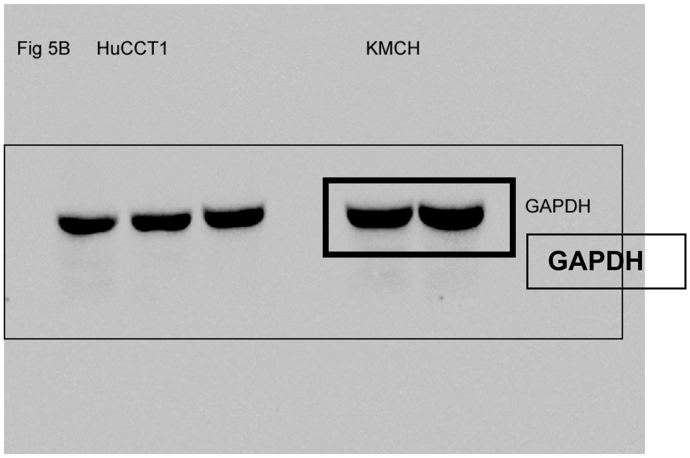

Fig 5b KMCH GAPDH (Low exposure)

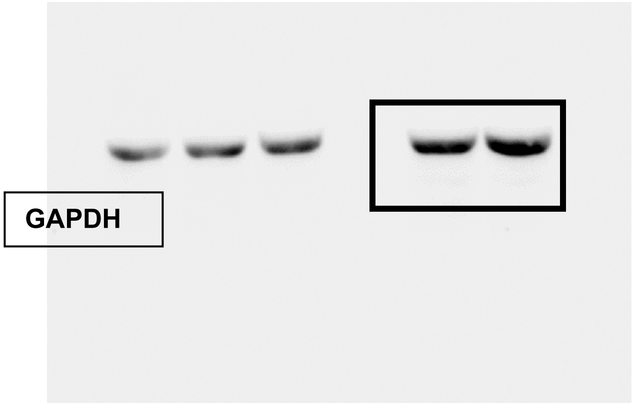

(thin lined black rectangles represent blot size).

Original Western blots for Figure 6B

Fig 6B CDK5

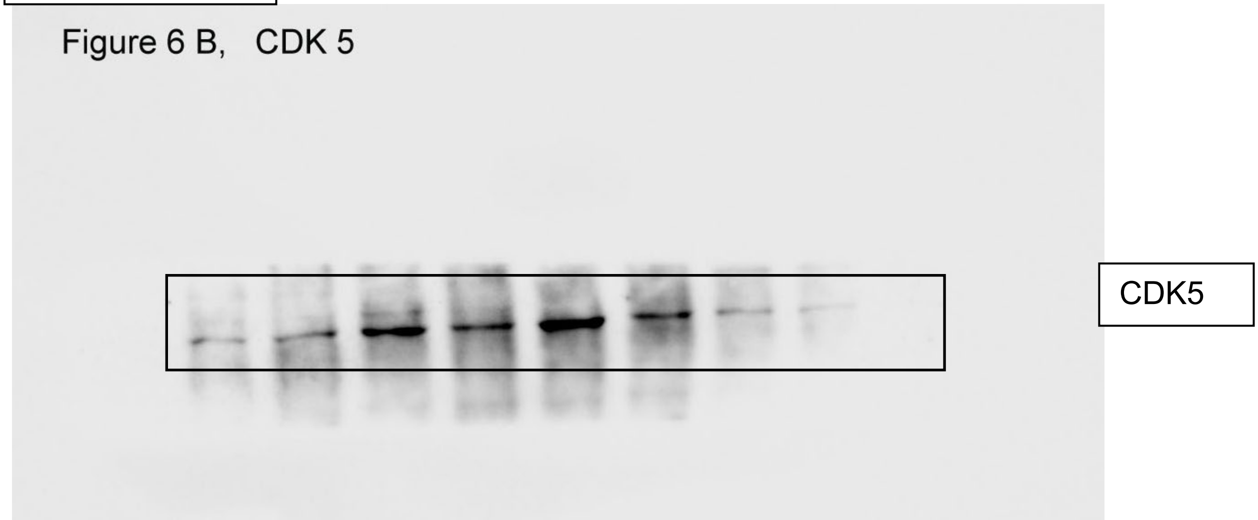

CDK5

Fig 6B CDK2

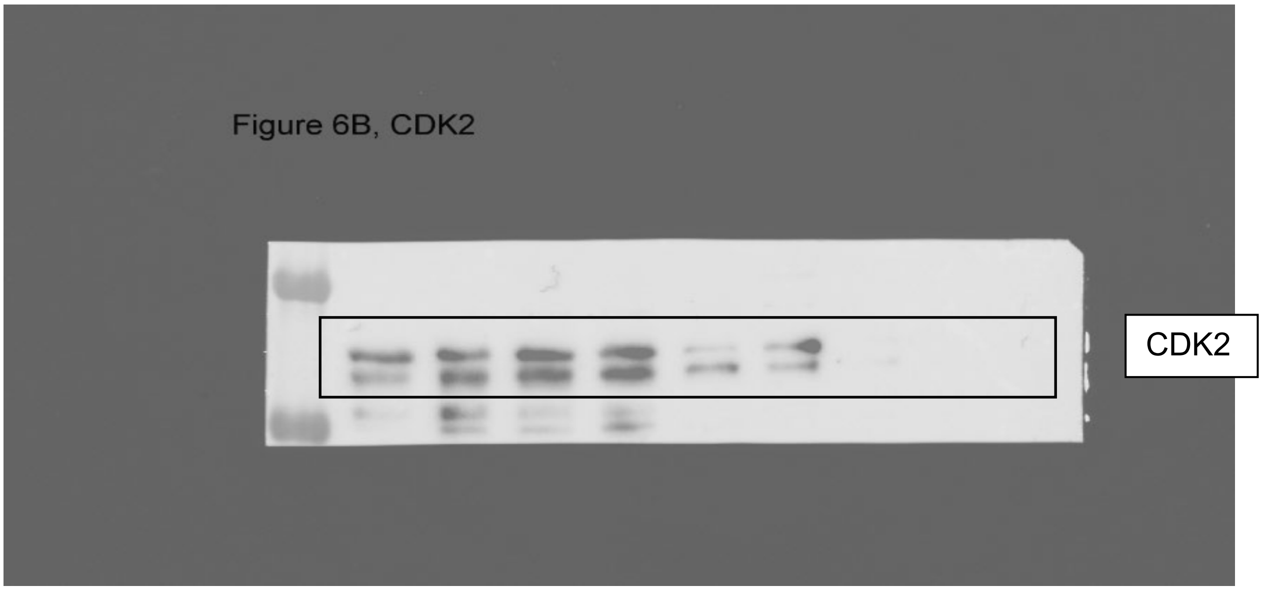

CDK2

Fig 6B CDK9, Lower band

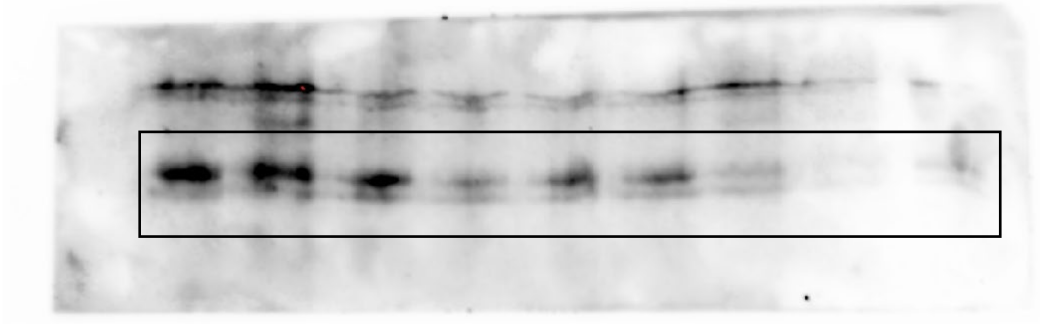

CDK9

Fig 6B BCL2

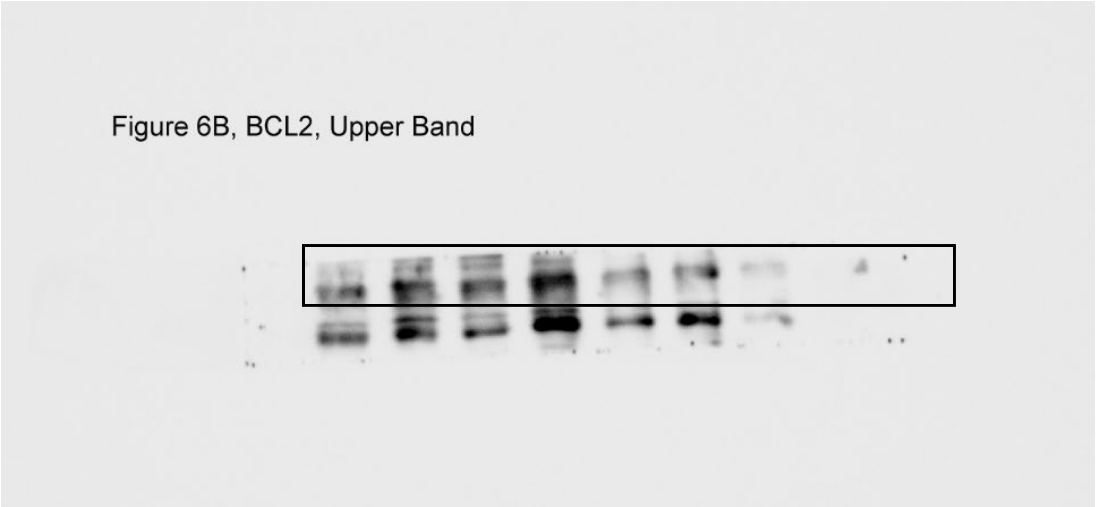

BCL2

Fig 6B Pro-PARP

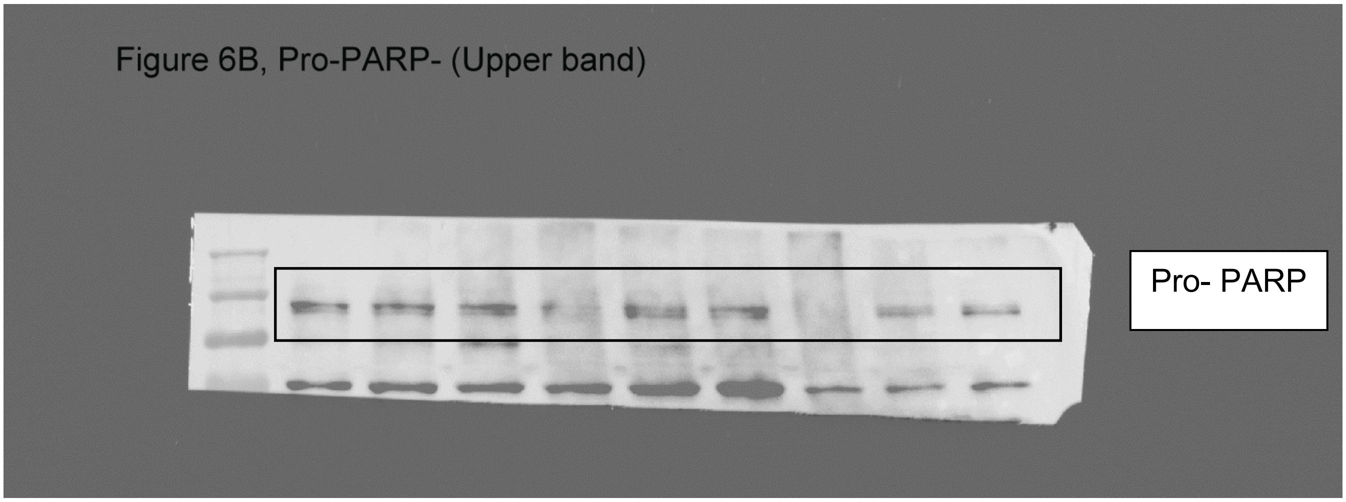

Pro- PARP

Fig. 6B. pRB (Ser807/811)

Low exposure

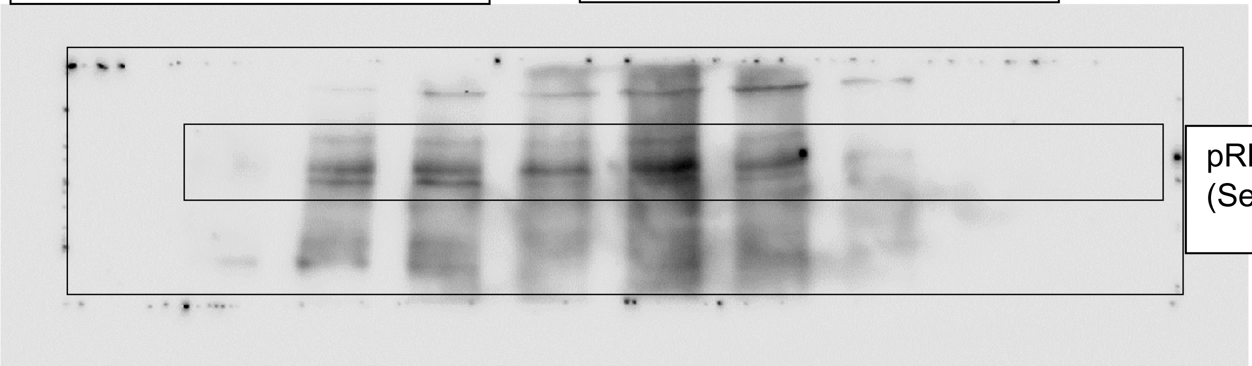

pRB (Ser807/811)

Fig. 6B. pRB (Ser807/811)

High exposure

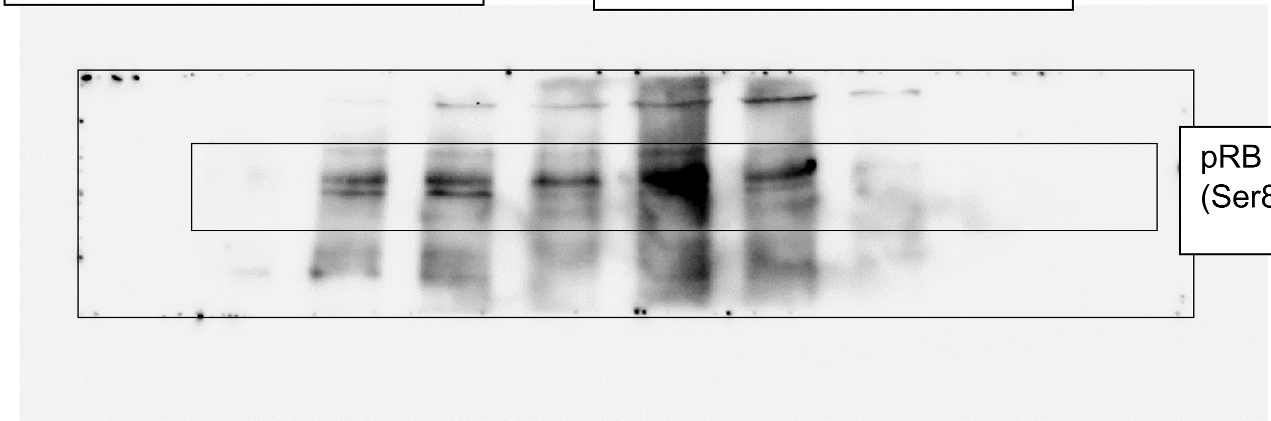

pRB (Ser807/811)

Fig 6B   BCL-XL

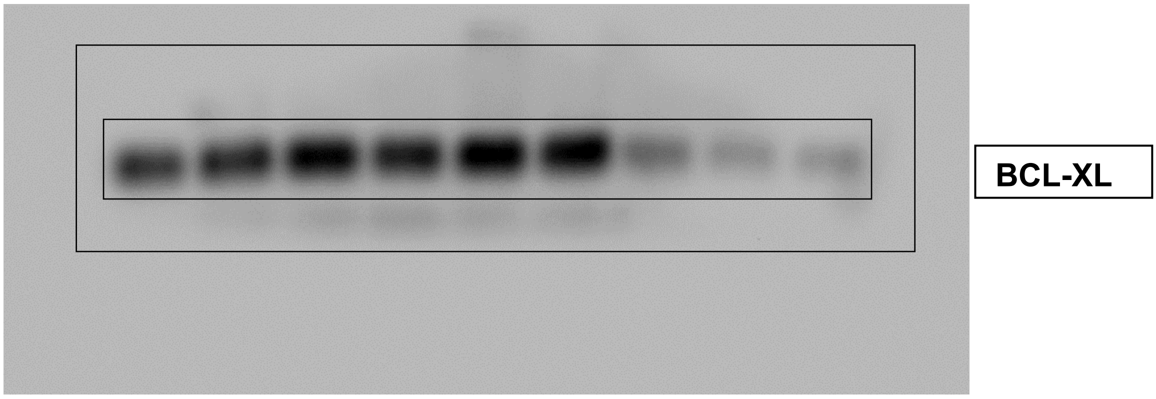

BCL-XL

Fig 6B GAPDH

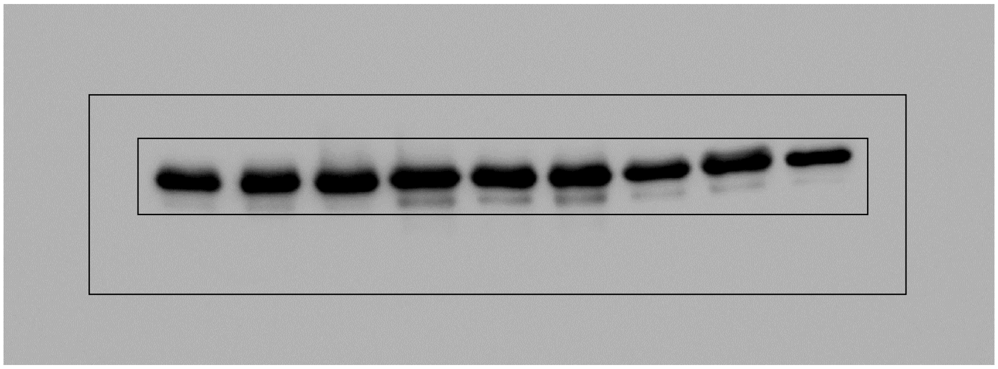

GAPDH

Supplemental Figure 1C

Original Western blots for Supplemental Figure 1C

GAPDH

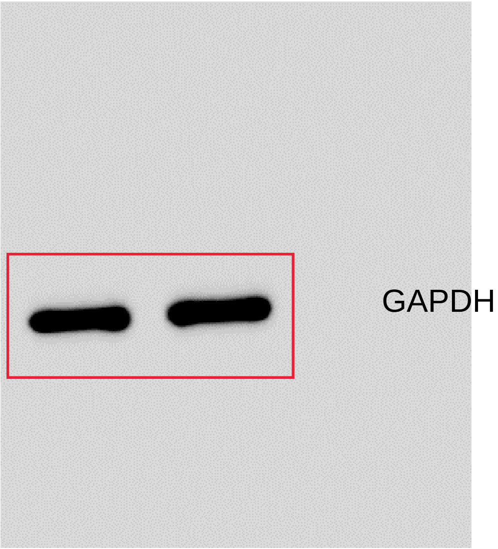

CDK4

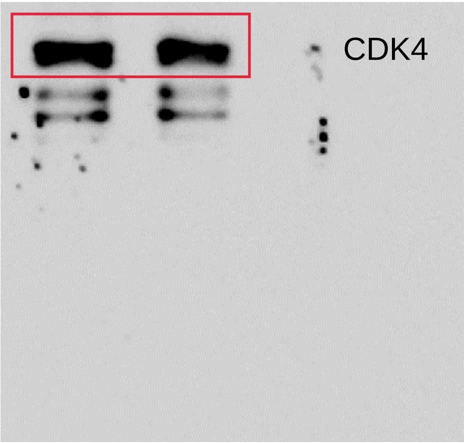

CDK 6

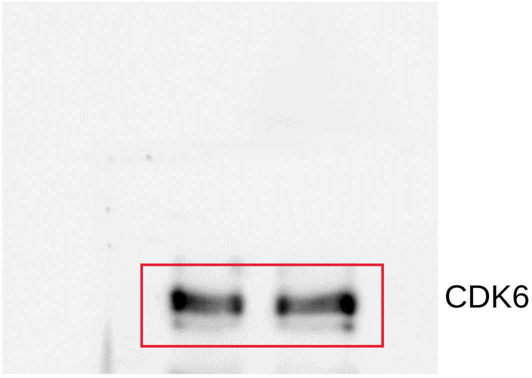

Supplement: Supplementary file 1 — Supplementary Information [file 41598_2020_75578_MOESM1_ESM.pdf]
